# Stress granule formation is regulated by signaling machinery involving Sch9/Ypk1, sphingolipids, and Ubi4

**DOI:** 10.7150/thno.98199

**Published:** 2025-01-06

**Authors:** Lihua Chen, Yuan Gao, Xinxin Hao, Xiaoxue Yang, Michelle Lindström, Shan Jiang, Xiuling Cao, Huisheng Liu, Thomas Nyström, Per Sunnerhagen, Beidong Liu

**Affiliations:** 1 Department of Chemistry and Molecular Biology, University of Gothenburg, S-413 90, Göteborg, Sweden.; 2 Guangzhou National Laboratory, Guangzhou, Guangdong, China.; 3 School of Biomedical Engineering, Guangzhou Medical University, Guangzhou, Guangdong, China.; 4 State Key Laboratory of Subtropical Silviculture, School of Forestry and Biotechnology, Zhejiang A&F University, Lin'an, Hangzhou, 311300, China.; 5 EATRIS Center for Large-scale cell-based screening, Department of Chemistry and Molecular Biology, University of Gothenburg, S-413 90, Göteborg, Sweden.

**Keywords:** stress granules, mammalian target of rapamycin (mTOR), ubiquitination, sphingolipids, phase separation

## Abstract

**Rationale:** Stress granules (SGs) are membraneless organelles that are formed in response to various stresses. Multiple cellular processes have been reported to be involved in SG formation. However, the signaling cascades that coordinate SG formation remain to be elucidated.

**Methods:** By performing two high-content imaging-based phenomic screens, we identified multiple signaling components that form a possible signal transduction pathway that regulates SG formation.

**Results:** We found that Sch9 and Ypk1 function in an early step of SG formation, leading to a decrease in intermediate long-chain base sphingolipids (LCBs). This further downregulates the polyubiquitin precursor protein Ubi4 through upregulating the deubiquitinase Ubp3. Decreased levels of cellular free ubiquitin may subsequently facilitate Lsm7 phase separation and thus trigger SG formation.

**Conclusion:** The signaling pathway identified in this work, together with its conserved components, provides valuable clues for understanding the mechanisms underlying SG formation and SG-associated human diseases.

## Introduction

Cytoplasmic stress granules (SGs) are membraneless organelles that temporarily assemble to facilitate rapid cessation of translation in response to stress conditions 1-9 or normal cellular processes such as G protein activation 10. SGs typically include a plethora of translation initiation components and nontranslating mRNAs, in addition to proteins that regulate mRNA function 1, 3, 11, 12. Accumulating evidence has shown that ribonucleoprotein particle (RNP) granules, including SGs, form through a process called liquid-liquid phase separation (LLPS). These highly conserved subcellular structures are tightly regulated and thought to form via LLPS of low-complexity protein domains 13-16. Moreover, RNAs can impact the phase separation of various RNA-binding proteins (RBPs) 17-19. In addition, cellular signaling and catalytic proteins have been shown to be sequestered in SGs 5, 20, 21. Dysregulation of SGs is connected to many pathologies, ranging from neurodegenerative diseases to cancers 22-28.

The formation of SGs affects cellular signaling pathways, thereby controlling cell fate or function 21, 24, 29-33. Accordingly, SG formation affects target of rapamycin (TOR) signaling, which coordinates cellular responses to stressful conditions such as nutritional deficiencies. A key component of the yeast TOR1 complex, Kog1 (raptor in mammalian cells), is transiently recruited to SGs upon heat shock or oxidative stress, thereby delaying the reactivation of TORC1 signaling or preventing mTORC1 hyperactivation-induced apoptosis 24, 29. However, whether TORC1/2 or any of their downstream components have active signaling roles in regulating SG formation is unclear.

The sphingolipid metabolism pathway has important roles in signal transmission and cell recognition, and disorders in this pathway affect neural tissues 34-36. The composition of sphingolipids may be altered due to changes under physiological conditions. For example, when yeast cells enter the stationary phase, there is a large increase in total free and sphingolipid-bound long chain bases, which are regulated mainly by intrinsic factors, such as Orm1 and Orm2, and serine palmitoyltransferase 37. In contrast, under glucose restriction, the amount of free long chain bases decreases 37. These results highlight the physiological role of long chain bases in stress resistance and survival. Furthermore, sphingolipid have been reported to mediate processing body (PB, another type of RNA granule) formation and translation initiation during heat stress 34. However, whether there is a link between the physiological role of sphingolipids and SG formation has not been determined.

Recent progress has highlighted the ubiquitin-based machinery that regulates SG formation. It has been shown that free ubiquitin, rather than ubiquitin-conjugated proteins, colocalizes to SGs and that free ubiquitin may alter SG protein interactions 38. In accordance, specific noncovalent ubiquitin binding may disrupt the LLPS of SG-residing proteins 39. The cellular ubiquitin pool comprises free ubiquitin and ubiquitin conjugates. Under normal conditions, the bulk of ubiquitin is controlled by three ubiquitin-fused ribosomal proteins, *RPL40A* (*UBI1*), *RPL40B* (*UBI2*), and *RPS31* (*UBI3*). In yeast, an additional gene, *UBI4,* accounts for the main source of *de novo* ubiquitin synthesis under various stresses, including heat stress, oxidative stress, starvation, and the stationary phase 40, 41, while the expression of the other three ubiquitin genes is suppressed during stress conditions 42. In addition to Ubi4, the yeast deubiquitinase Ubp3 (human USP10) has been found to be a constituent and regulator of SGs 43-45.

In this work, we report a signaling pathway that controls the formation of SGs under 2-deoxyglucose (2-DG) treatment. Using an imaging-based phenomic screen, we found that Sch9 and Ypk1 are downstream effectors of TORC1/2 complexes involved in the regulation of SG formation. The TORC1/2-Sch9/Ypk1 signaling cascade further decreases the levels of major long-chain base sphingolipids (LCBs) in the cell. This subsequently downregulates the expression of the ubiquitin-proteasome system (UPS) component Ubi4, in part by upregulating the deubiquitinase Ubp3. In addition, we found that a SG initiation factor - Lsm7 - is located downstream of the Ubi4-Ubp3 signaling. Ubp3 positively regulates Lsm7 phase separation, thereby triggering SG formation.

## Results

### Essential components involved in SG formation

**Figure 1:**
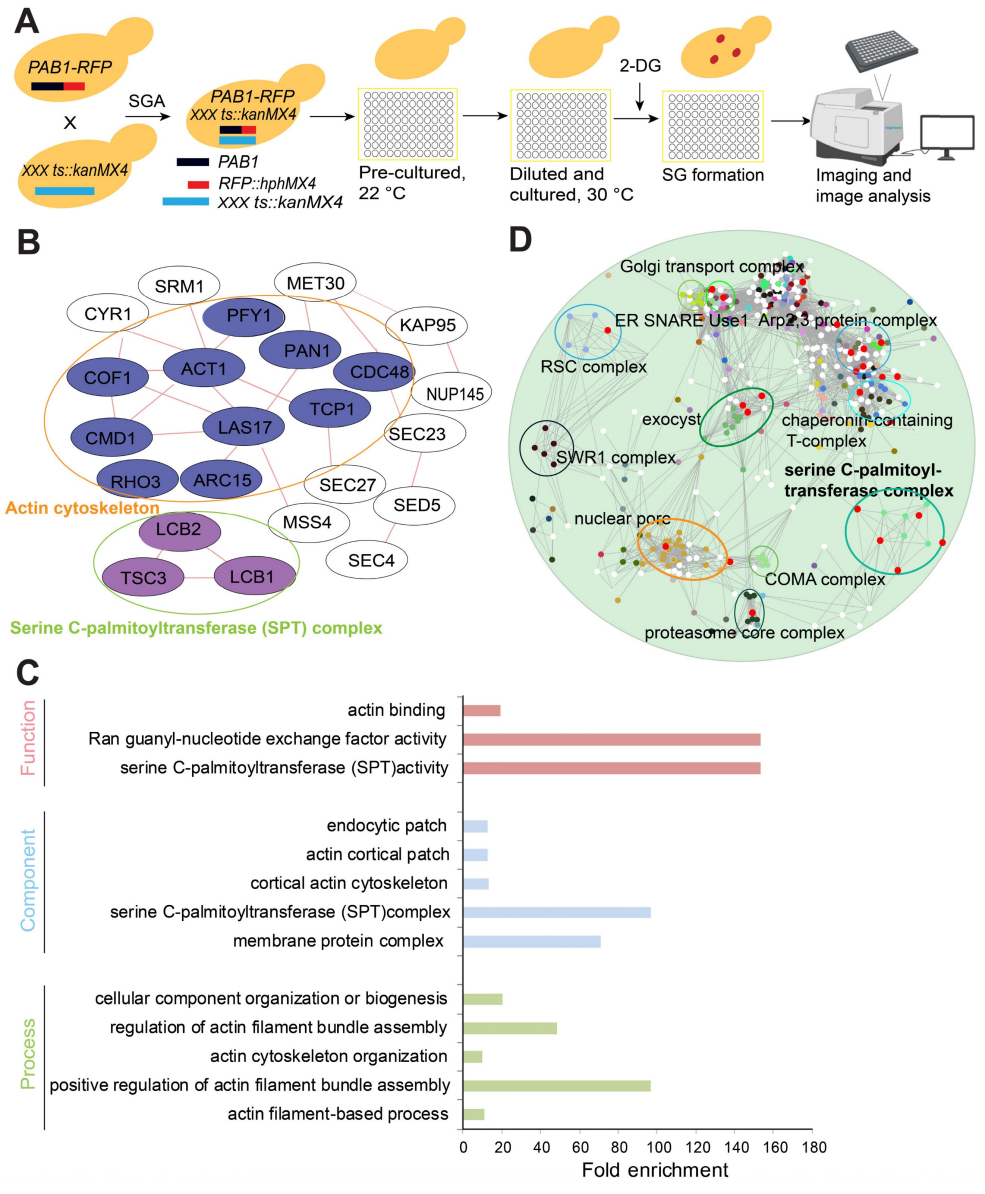
** A high-content screening-based study identified the serine C-palmitoyltransferase complex as a major regulator of SG formation. A,** The workflow for screening SG formation (Pab1-RFP foci are represented by red dots) in response to temperature-sensitive (ts) alleles of essential genes under 2-DG treatment. **B,** Network analysis of the genes whose ts alleles showed increased SG formation. The genes were grouped into modules based on their known physical interactions (red lines) and published cellular component information.** C,** Functional enrichment analysis of the ts alleles associated with increased SG formation. Mutants with confirmed SG phenotypes were analyzed for enrichment of the GO biological process, function and component categories. Enriched groups were scored by comparison to the essential gene temperature-sensitive allele collection using a cutoff of P < 0.05.** D,** A genetic interaction profile similarity network analysis (annotated with protein complex) for the ts alleles that showed increased SG formation.

To identify essential SG signaling components, we systematically quantified SG formation using the yeast essential gene temperature sensitive (ts) allele collection 46. We chose 400 mM 2-DG as the SG inducer since 2-DG can induce robust and steady SG formation 47. The library for the SG formation screen was constructed by crossing a query strain (Y7092) bearing a red fluorescent protein-tagged Pab1 (Pab1-RFP) with the ts allele collection by using the SGA method 48 (Figure 1A). After manual confirmation, we obtained 31 mutants with significantly increased SG formation. These confirmed hits were analyzed for physical interaction networks using the GeneMANIA plug-in for Cytoscape (Cytoscape v3.2.1) 49. The network analysis indicated that the genes of the mutants with increased SG formation were enriched for products that physically interact with each other. The interacting proteins included those with cellular functions such as (1) serine C-palmitoyltransferase activity (sphingolipid biosynthesis) and (2) actin cytoskeleton/binding (Figure 1B).

The top hits were also analyzed using the Gene Ontology Term Finder 50 for enrichment of Gene Ontology (GO) biological processes, molecular functions, and cellular component categories via comparisons with a background set of genes (TS-V5) 46. The enriched cellular functions were enriched for (1) serine C-palmitoyltransferase activity (sphingolipid biosynthesis), (2) actin binding, and (3) Ran guanyl-nucleotide exchange factor activity; the enriched cellular components were (1) serine C-palmitoyltransferase complex, (2) endocytic patch, (3) actin cortical patch, (4) cortical actin cytoskeleton, and (5) membrane protein complex; and the enriched biological processes were (1) cellular component organization or biogenesis, (2) regulation of actin filament bundle assembly, (3) positive regulation of actin filament bundle assembly, (4) actin cytoskeleton organization, and (5) actin filament-based process (Figure 1C).

Furthermore, we performed a genetic interaction profile similarity network analysis (annotated with protein complex) for the ts alleles that displayed increased SG formation. The results showed that these ts alleles were enriched in the following complexes: serine C-palmitoyltransferase complex, Arp2,3 protein complex, exocyst, proteasome core complex, nuclear pore, and Golgi transport complex; or interacted with these complexes: chaperonin-containing T-complex, COMA complex, SWR1 complex, RSC complex, nuclear pore, and ER SNARE Use1 (Figure 1D). From these analyses, ts mutants of the serine palmitoyltransferase complex (*lcb1-4, lcb2-16* and *tsc3-2*) were identified as a consistent significantly enriched functional group from our screen.

From the screen, we also identified 47 ts mutants with significantly decreased SG formation. Gene Ontology (GO) analysis indicated that gene mutants associated with decreased SG formation were significantly enriched for the following cellular components: (1) TOR signaling, (2) proteasomal core complex, and (3) DNA polymerase complex (Figure S1A). The functional enrichment analysis of the ts alleles associated with decreased SG formation revealed enrichment of “cellular function” GO terms (1) helicase activity, (2) protein kinase activator activity, and (3) kinase activator activity; “component” GO terms (1) delta DNA polymerase complex, (2) proteasome core complex, (3) cytosolic proteasome complex, (4) proteasome core complex, beta-subunit complex, and (5) TOR complex; and “process” GO terms (1) regulation of cell growth, (2) nucleocytoplasmic transport, (3) establishment or maintenance of actin cytoskeleton polarity, (4) ribonucleoprotein complex biogenesis, (5) proteasomal ubiquitin-independent protein catabolic process, and (5) TOR signaling (Figure S1B).

### LCBs as regulators of SG formation

**Figure 2:**
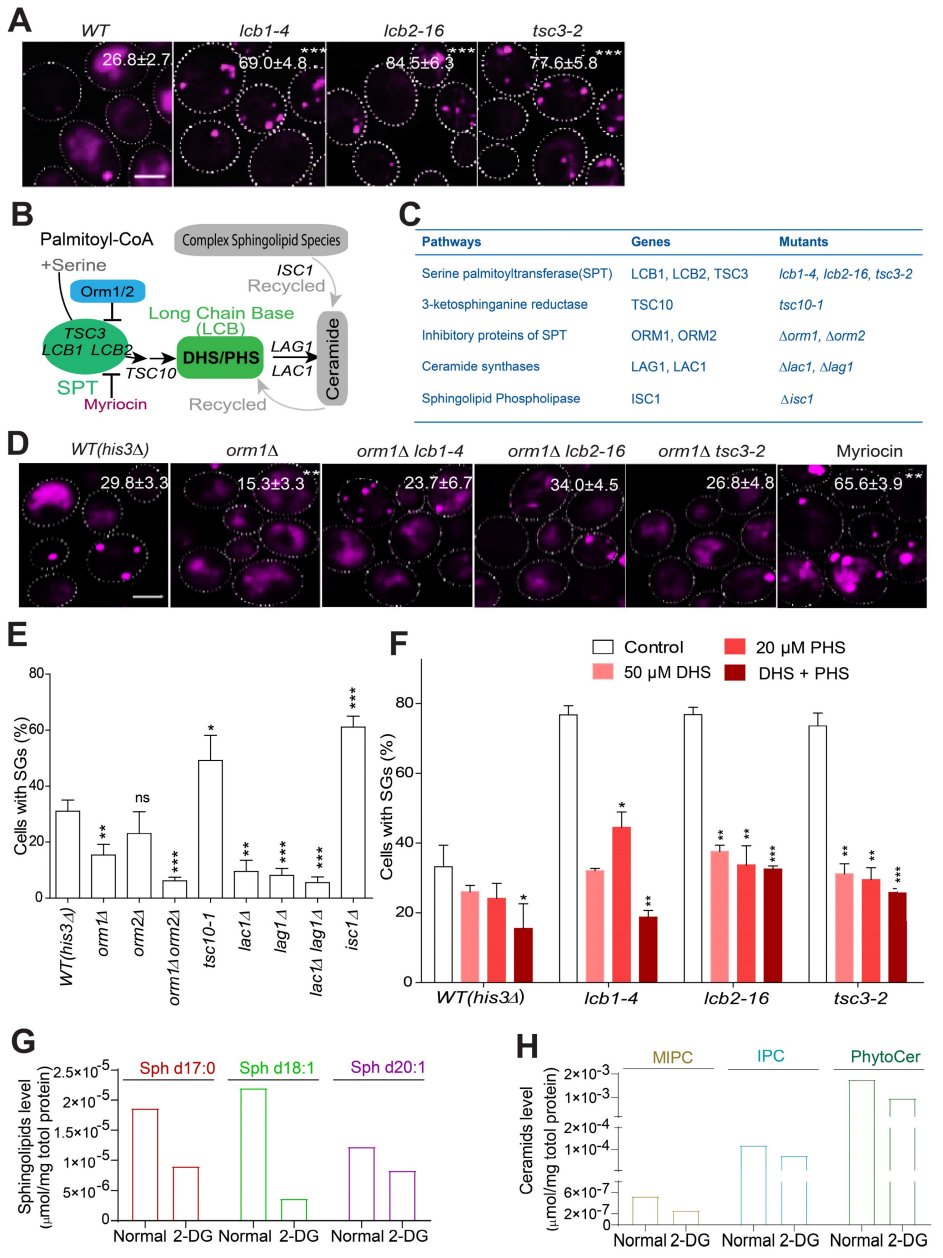
** LCBs as regulators of SG formation. A,** Mutants of the serine palmitoyltransferase (SPT) complex (*LCB1*, *LCB2*, and *TSC3*) significantly increase SG formation. The values represent the percentage of cells with SGs and are shown as the means ± S.D.s. Six clones were examined for each strain, and >300 cells were analyzed for each colony. The scale bars indicate 2 µm, * P < 0.05; ** P < 0.01; *** P < 0.001 (unpaired two-tailed Student's t test).** B,** A schematic diagram showing how the key sphingolipid metabolism pathway intermediates, LCB sphingolipids, are regulated by multiple genes/pathways. **C,** The genes related to sphingolipid metabolism pathway and the corresponding mutants examined in this study were shown. **D,** Deletion of *ORM1* decreased SG formation in the WT strain; suppressed SG induction in the *lcb1-4*, *lcb2-1*, and *tsc3-2* mutants; and the inhibition of the SPT complex by myriocin increased SG formation. The values represent the percentage of cells with SGs and are shown as the means ± S.D.s. Six clones were examined for each strain, and >300 cells were analyzed for each colony. The scale bars indicate 2 µm, * P < 0.05; ** P < 0.01; *** P < 0.001 (one-way ANOVA followed by Dunnett's test).** E,** SG formation was increased in the* tsc10-1* and *isc1∆* mutants and was decreased in the *orm1∆, orm1∆orm2, ∆lac1∆*, *lag1∆*, and* lac1∆lag1∆* mutants. Same as in (**D**)**. F,** Exogenous DHS, PHS, and their combination (50 μM DHS + 20 μM PHS) can restore the increased SG formation in the SPT ts mutants.** G-H,** The major long chain-base sphingolipids (dihydrosphingosine (C17 base), sphingosine (d18:1), sphingosine (d20:1)) and ceramides (MIPC, IPC, PhytoCer) decreased in WT (*his3Δ*) cells after 2-DG treatment (2 h). 7 OD WT cells were collected after 2 h of 400 mM 2-DG treatment in SD medium and subject to lipidomics analysis. One representative experiment was shown.

Among the increased SG formation hits, our screen identified three ts mutants of the serine palmitoyltransferase complex (*lcb1-4*, *lcb2-16* and *tsc3-2*), which formed a consistently enriched group according to different analyses (Figure 1B-D). The increased SG-formation phenotype of these mutants was also among the top groups of hits (Table S1). This complex is highly conserved across eukaryotic cells and is the first rate-limiting step during sphingolipid biosynthesis. Therefore, we hypothesized that the sphingolipid metabolism pathway may play a role in the regulation of SG formation. We first manually confirmed the increased SG phenotype of these mutants (Figure 2A), and showed that their increase in SG formation was not due to enhanced expression of SG marker Pab1-RFP (Figure S2A).

The sphingolipid synthesis pathway is known to be regulated by multiple cellular components, including Orm1/2 and Lac1/Lag1, and the recycling pathway coordinated by Isc1 (Figure 2B) 51, 52. To better understand how sphingolipid synthesis pathway components influence SG formation, we tested available mutants of these regulators (Figure 2C). First, we found that the derepression of serine palmitoyltransferase (SPT) by the deletion of *ORM1* decreased SG formation compared to that in the control strain (*his3∆*) (Figure 2D), while the time SGs took to fuse was not changed between the two strains (Figure S2B). And this deletion further dampened SG induction in the *lcb1-4*, *lcb2-1*, and *tsc3-2* mutants (Figure 2D). Since *ORM1* has the paralog *ORM2,* we tested the* ORM2* single mutant, in addition to the *orm1∆ orm2∆* double mutant, for its SG phenotype. The phenotype of the *orm2∆* single mutant was not significant (although a trend toward a decrease in SG formation was observed), but the *orm1∆ orm2∆* double mutant exhibited a significant additive effect on decreasing SG formation (Figure 2E)*.* Accordingly, myriocin, a drug that primarily inhibits the SPT complex and leads to the depletion of sphingolipids such as dihydrosphingosine (DHS) 53, greatly increased SG formation (Figure 2D). Third, we found that the *tsc10-1* mutant (Tsc10 is a component that catalyzes the second step in phytosphingosine synthesis) exhibited a similar phenotype to that of the SPT mutants, and SG formation was decreased in the *lac1∆*, *lag1∆*, and* lac1∆lag1∆* mutants but strongly increased in the *isc1∆* mutant (Figure 2E). Previous research has demonstrated that phytosphingosine (PHS) and DHS are increased in *lac1∆* and* lag1∆* cells 54 . Moreover, in the *isc1∆* mutant, the levels of DHS and PHS might decrease because Isc1 is responsible for the recycling of ceramides to generate DHS and PHS 51, 55. We then hypothesized that DHS and PHS may be important signaling components that negatively regulate SG formation. Indeed, we found that exogenous DHS, PHS, or their combination could suppress the increase in SG formation in the SPT ts mutants (Figure 2F). In support of this, the major LCBs and ceramides were decreased in the wild-type strain upon 2-DG treatment (Figure 2G-H), and the protein expression level of the SPT component Tsc3 decreased upon 2-DG treatment (Figure S2C). Meanwhile, we used another SG marker eIF4G1 56, 57 to further verify our findings. The data showed that the foci formation of eIF4G1 was similar to that of Pab1 under myriocin or DHS + PHS treatment (Figure S2D).

To explore if the signaling cascade here reported is a common regulator of SGs formation in yeasts, we applied other stress inducers, including glucose depletion and oxidative stress. It was found that the SPT ts alleles had similar SGs phenotype under glucose depletion (Figure S2E) and arsenite treatment (Figure S2F) as 2-DG treatment, which suggest the signaling cascade we elucidated can be conserved in yeast cells.

**Figure 3:**
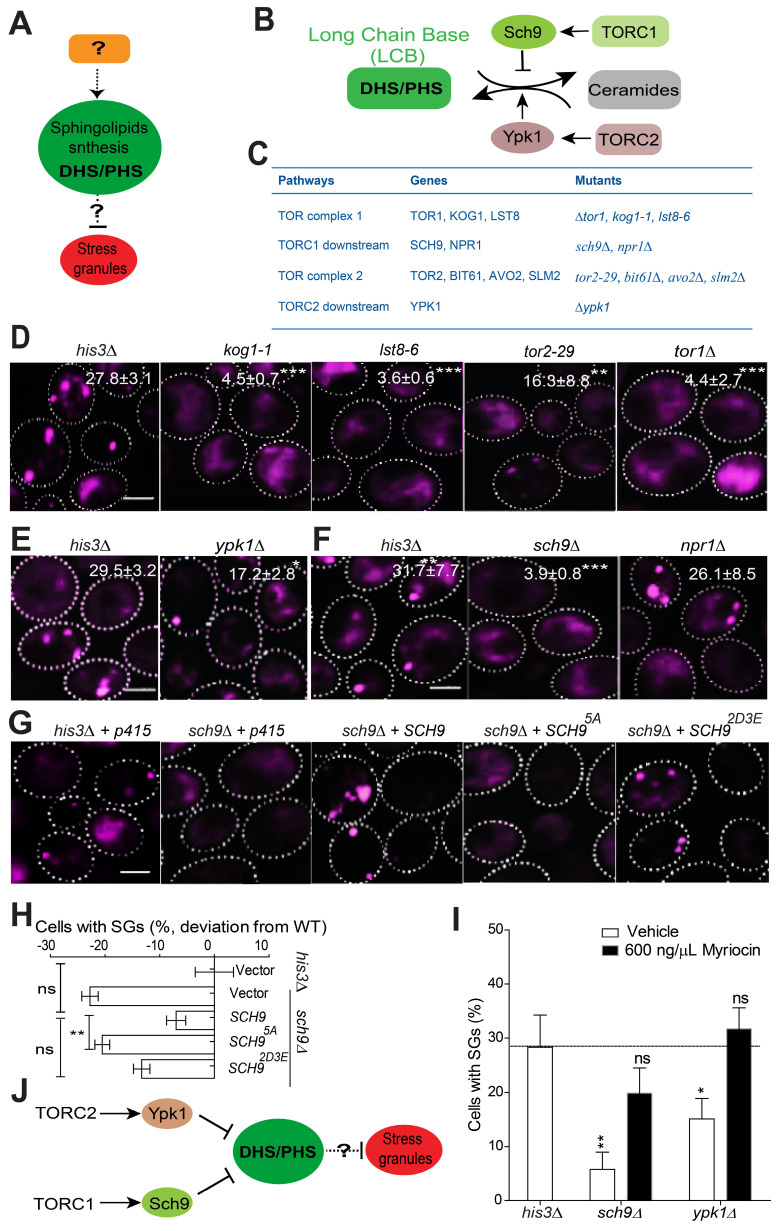
**TORC1-Sch9 and TORC2-Ypk1 function upstream of LCB sphingolipids. A,** A schematic diagram showing that LCBs regulate SG formation; however, the upstream regulators and downstream effectors involved are unknown.** B,** A schematic diagram showing the regulation of the sphingolipid metabolism pathway by the TORC1/2 signaling components identified from our screen in budding yeast.** C,** The genes related to TORC1/2 signaling pathway and the corresponding mutants examined in this study were shown. **D,** TORC1/2 ts mutants (*kog1-1, lst8‑6, tor2-29*) and the null mutant *tor1∆* exhibit significant defects in SG formation. The values represent the percentage of cells with SGs and are shown as the means ± S.D.s. Six clones were examined for each strain, and >300 cells were analyzed for each colony. The scale bars indicate 2 µm, ** P < 0.01; *** P < 0.001 (unpaired two-tailed Student's t test).** E,** Deletion of the TORC2 downstream effector *YPK1* results in a significant defect in SG formation. As in (**C**), * P < 0.05 (unpaired two-tailed Student's t test).** F,** Deletion of *SCH9* results in a clear defect in SG formation, while deletion of *NPR1* has no effects. As in (**D**), *** P < 0.001 (unpaired two-tailed Student's t test).** G-H,** The inactive form of *SCH9^5A^* fails to complement the SG phenotype of the *sch9∆* mutant, but the constitutively active mutant* SCH9^2D3E^* can restore SG formation. The values represent the percentage of cells with SGs and are shown as the means ± S.D.s. Six clones were examined for each strain, and >300 cells were analyzed for each colony. The scale bar indicates 2 µm, ** P < 0.01 (*sch9∆* + *SCH9^5A^* vs*. sch9∆* + *SCH9*); ns, not significant (WT + vector vs*. sch9∆* + *SCH9*; *sch9∆* + *SCH9* vs*. sch9∆* + *SCH9^2D3E^*) (one-way ANOVA followed by Tukey's test).** I,** The addition of myriocin reversed the defects in SG formation in the *sch9∆* and* ypk1∆* mutants. Same as in (**G**).** J,** A diagram summarizing the current data and suggesting that TORC1-Sch9 and TORC2-Ypk1 function upstream of LCB sphingolipids in the regulation of SG formation.

These data suggested that DHS and PHS function as signaling components that negatively regulate SG formation and further motivated us to search for upstream regulators and downstream effectors of DHS and PHS in the SG formation process (Figure 3A).

### Sch9 and Ypk1 function upstream of LCBs

Sch9 and Ypk1 are known to be the main components that regulate sphingolipid metabolism (Figure 3B-C; summarized from Kitagaki *et al.* and Muir* et al.*) 51, 52. Ypk1 and Sch9 function as key regulators of sphingolipid metabolism and maintain low levels of LCBs and LCB phosphates (LCBPs) and high levels of ceramides 51, 55. Given that Sch9 and Ypk1 are known downstream effectors of TORC1 and TORC2, respectively, we hypothesized that Sch9 and Ypk1 may serve as upstream regulators of LCBs to govern SG formation together with the TORC1/2 complex.

The TORC1 complex has been implicated in SG formation. In mammalian cells, under heat shock or oxidative stress, the components of mTORC1 are recruited into SGs by astrin 24, 29. Moreover, the mTOR effector kinases S6 kinase 1 (S6K1) and S6 kinase 2 (S6K2) also localize to SGs in human cells under mild oxidative stress 58. TORC2 mediates the heat stress response in *Drosophila* by promoting the autonomous formation of SGs 59. We first verified whether the TORC1/2 complex plays an active role in regulating SG formation under 2DG-induced stress conditions. Our screening data and further manual confirmation showed that the TORC1/2-related mutants *kog1-1*, *lst8-6, tor2-29* and *tor1∆* exhibited significant defects in SG formation (Figure S1A-B and Figure 3D) without altering the expression of the SG marker Pab1-RFP (Figure S3A). These findings indicated that TOR complexes play a functional role in regulating SG formation under 2-DG-induced stress conditions. We then evaluated the SG phenotype in the absence of other available TORC1/2 core components, including Avo2, Bit61, and Slm2. All these mutants exhibited a substantial decrease in SG formation (Figure S3B), and their SG-defective phenotypes were not due to a decreased expression level of the SG marker Pab1-RFP (Figure S3C). Next, we found that the Kog1-GFP, Lst8-GFP, and Bit61-GFP signals did not co-localize with SGs (Figure S3D). This indicated that their SG phenotypes do not result from a reduction in their gene products as SG components.

TORC2 is known to control sphingolipid homeostasis through Ypk1 51, 60. We found that SG formation decreased in *ypk1∆* strains (Figure 3E). These data, together with the findings that SG formation is decreased in other core TORC2 component mutants (*tor2-29*, *avo2∆, bit61∆,* and* slm2∆*) (Figure S3B-C), indicate that Ypk1 may work downstream of TORC2 in the signal transduction process during SG formation. However, given that the SG phenotype of *ypk1*∆ strains is weaker than that of *avo2∆, bit61∆,* and* slm2∆* strains, this indicates that TORC2 signaling may involve other effectors in addition to *YPK1* that regulate 2-DG-induced SG formation.

Yeast TORC1 functions mainly through Npr1 and Sch9 61, 62. We found that *sch9∆* cells displayed an SG formation defect, while the *npr1∆* mutation had no clear effect (Figure 3F). We further found that the inactive form *SCH9^5A^* failed to complement the SG phenotype of the *sch9∆* mutant but that the constitutively active mutant* SCH9^2D3E^* could (Figure 3G-H).

To clarify the link between *SCH9/YPK1* and LCBs, we added myriocin to the *sch9∆* and *ypk1∆* mutants to determine whether inhibiting LCB synthesis could reverse the SG phenotype. As shown in Figure 3I, the addition of myriocin reversed the defects in SG formation; this result indicates a connection between* SCH9/YPK1* and LCBs in the regulation of SG formation. Again, we showed that the SG phenotype of *sch9∆* and *ypk1∆* mutants is similar under arsenite (Figure S3E) as under 2-DG treatment, suggesting that the signaling cascade is a common regulator of SG formation in yeast cells.

Taken together, these results suggest that Sch9 and Ypk1 work upstream of LCB sphingolipids to regulate SG formation (Figure 3J). However, the factors located downstream of LCBs in the SG formation control pathway remain to be determined.

### Reduced LCBs decrease Ubi4 expression levels and thereby enable SG formation

**Figure 4:**
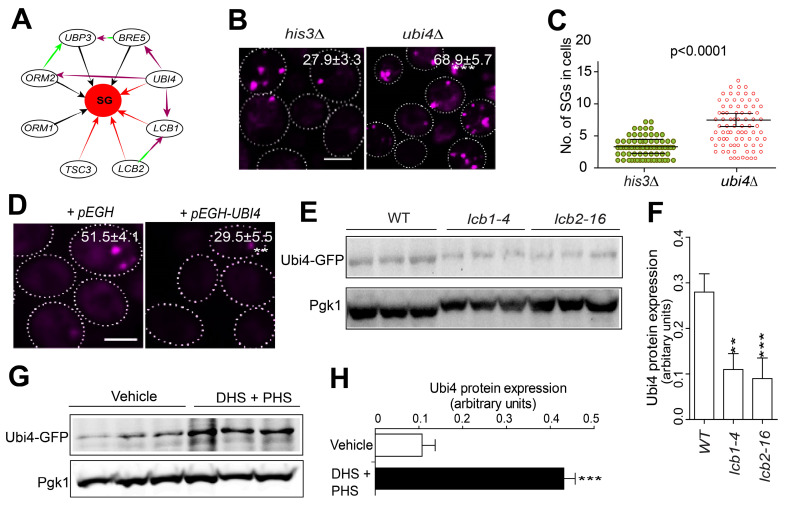
** Ubi4 suppresses SG formation. A,** Interaction network analysis of the SPT complex genes *LCB1*, *LCB2*, and *TSC3* (dark lines indicate a reduction in SG formation in the null mutants, and red lines indicate the induction of SG formation in the null mutants). The green lines indicate physical interactions, and the purple lines indicate genetic interactions. **B,** Deletion of *UBI4* triggers SG formation. Eight clones were examined for each strain, and >300 cells were analyzed for each colony. The scale bar indicates 2 µm, *** P < 0.001 (unpaired two-tailed Student's t test).** C,** Deletion of *UBI4* increases the number of SGs in the cell. Six clones were examined for each strain, and ~90 SG-containing cells were analyzed for each colony. One representative result is shown. The values are the numbers of SGs, and the P values are from unpaired two-tailed Student's t tests.** D,** Overexpression of *UBI4* decreases SG formation. Six clones were examined for each strain, and ~90 SG-containing cells were analyzed for each colony. One representative result is shown, and the scale bar indicates 2 µm. The values are the numbers of SGs, and the P values are from unpaired two-tailed Student's t tests.** E-F,** Ubi4-GFP (C-terminally GFP-tagged) expression is downregulated in *lcb1-4* and *lcb2-16*. One representative result with three different clones for each strain is shown. The values are presented as the means of arbitrary units (the intensity of the target bands was normalized to the Pgk1 level) for each clone.** G-H,** Ubi4-GFP expression was markedly increased by exogenous LCBs (25 µM DHS + 10 µM PHS or DHS + PHS). Same as in (**D-E**).

To search for components that work downstream of the sphingolipid pathway in regulating SG formation, we isolated all currently known interactors of the sphingolipid metabolism-related genes that were identified from our screen. We found that the sphingolipid-related gene *LCB1* has a genetic interaction with the UPS gene *UBI4* 63, 64 (Figure 4A)*.* Additionally, the *ORM2* gene, which inhibits *LCB1/2,* also genetically interacts with *UBI4 65*, and the protein products of *ORM2* and *UBP3* physically interact 66 (Figure 4A). Furthermore, *UBI4* is known to be induced by starvation and other stressors 40.

Following these clues, we wondered whether Ubi4 might act downstream of the sphingolipid pathway in SG formation signaling. We found that the deletion of *UBI4* triggered SG formation (Figure 4B-C). Consistently, overexpression of *UBI4* strongly suppressed SG formation (Figure 4D). Meanwhile, the Pab1-RFP protein expression was not changed in the Ubi4 deletion or overexpression strains (Figure S4). We further evaluated the relationship between SPT components and *UBI4* by investigating the Ubi4 protein expression level in the WT and the SPT ts mutants. We found that Ubi4 expression was significantly downregulated in *lcb1-4* and *tsc3-2* (Figure 4E-F). This finding suggested that *UBI4* could work downstream of the SPT complex in SG formation signaling. However, further evidence is needed to clarify how the SPT complex influences *UBI4* during this process. To address this question, we investigated the expression level of Ubi4 in cells exposed to the two main products of the sphingolipid synthesis pathway, DHS and PHS, and found that the level of Ubi4 was significantly increased (Figure 4G-H). This result showed that the SPT complex potentially regulates *UBI4* through influencing its downstream products DHS and PHS, and the cellular levels of DHS and PHS positively regulate the expression level of Ubi4. Taken together, these results suggest that Ubi4 may function as a downstream target of LCBs in the regulation of SG formation.

### LCBs enhance Ubi4 expression through suppressing Ubp3

**Figure 5:**
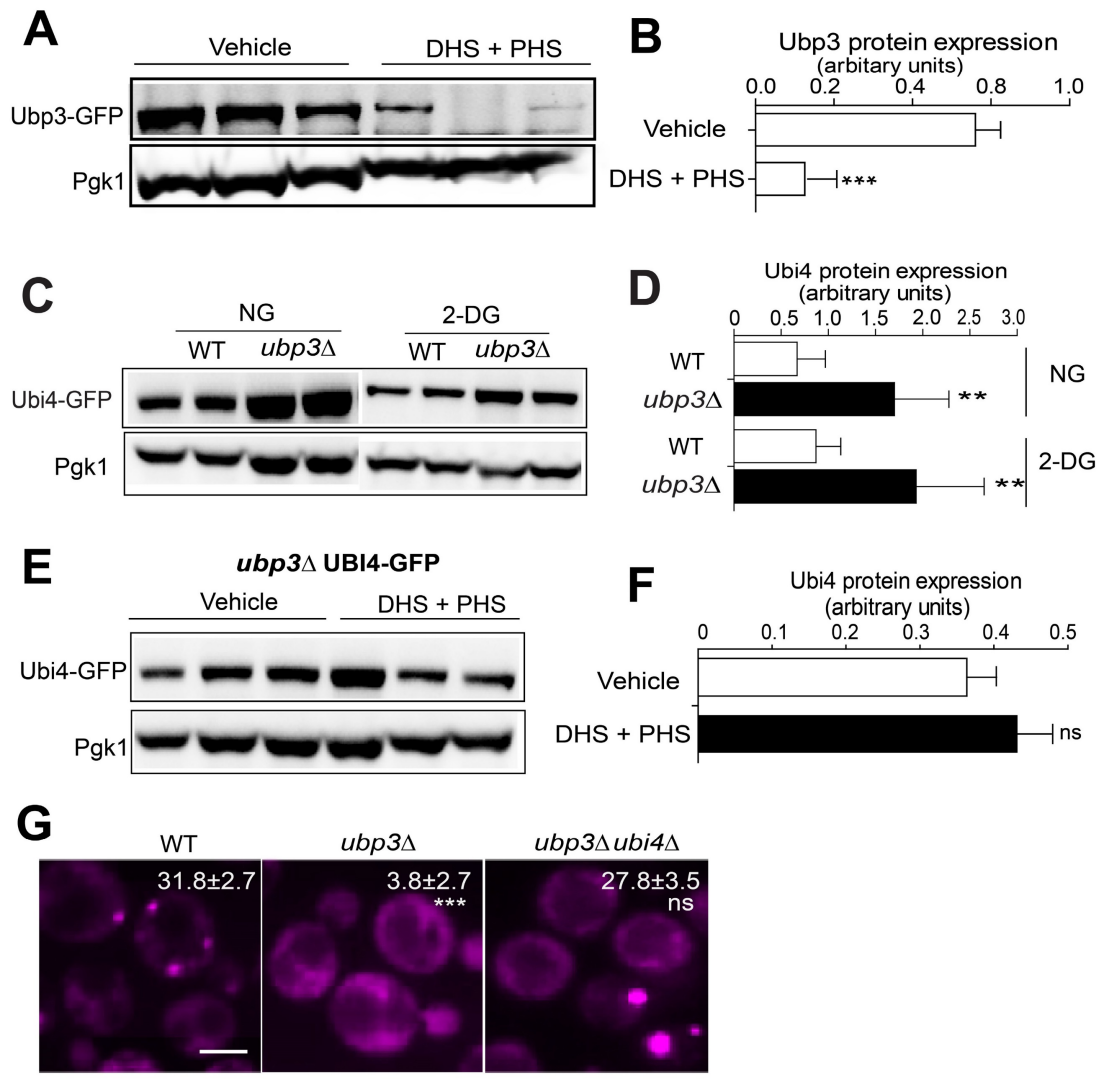
** LCBs upregulate Ubi4 expression through suppressing Ubp3. A-B,** Ubp3-GFP (C-terminally tagged with GFP) expression was downregulated by exogenous LCBs (25 µM DHS + 10 µM PHS or DHS + PHS). One representative result with three different clones for each strain is shown. The values are the means of the arbitrary units in (**B**) (the intensity of the target bands was normalized to the Pgk1 level) for each clone. *** P < 0.001 (unpaired two-tailed Student's t test).** C-D,** The Ubi4-GFP expression level increased when Ubp3 was deleted. One representative result with two different clones for each strain is shown. Same as in (**A-B**). ** P < 0.01 (unpaired two-tailed Student's t test). NG, normal glucose; DG, 400 mM 2-DG. **E-F,** The Ubi4-GFP expression level was not changed by exogenous LCBs (25 µM DHS + 10 µM PHS or DHS + PHS) when *UBP3* was deleted. One representative result with two different clones for each strain is shown. Same as in (**A-B**). ns, not significant (unpaired two-tailed Student's t test).** G,** Deletion of *UBI4* did not enhance SG formation in the absence of Ubp3. The values represent the percentage of cells with SGs and are shown as the means ± S.D.s. Six clones were examined for each strain, and >300 cells were analyzed for each colony; the scale bar indicates 2 µm. *** P < 0.001 (*ubp3∆* vs WT); ns, not significant (*ubp3∆ubi4∆* vs WT) (one-way ANOVA followed by Tukey's test).

The next question is how LCBs regulate Ubi4. To explore possible intermediators that signal from LCBs to Ubi4, we first surveyed published databases and found that Ubp3 can genetically interact with Ubi4 67, 68. The yeast Ubp3 is an mRNA-binding ubiquitin-specific protease that cleaves ubiquitin fusions 69, supported by its cofactor Bre5. The catalytic activity of Ubp3 is essential for SG assembly in yeast 45. Moreover, USP10, the mammalian homolog of Ubp3, has been found to be a constituent and regulator of SGs 43, 44. These findings motivated us to explore the relationship between Ubp3 and Ubi4 in the context of SG formation. First, we examined whether Ubp3 could also be regulated by LCBs. Upon the addition of exogenous DHS + PHS, Ubp3 protein expression was significantly downregulated (Figure 5A-B). Second, myriocin could not induce SG formation once *UBP3* was deleted (Figure S5A), and the SG phenotypes of the SPT ts alleles (*lcb1-4*, *lcb2-16* and *tsc3-2*) and *isc1* null mutant strains were abolished by *UBP3* deletion (Figure S5B). These data suggested that Ubp3 works downstream of the sphingolipid metabolism pathway in SG formation regulation and that the expression level of Ubp3 was negatively regulated by the level of DHS and PHS in the cell.

Subsequently, we determined whether Ubp3 could directly influence the expression of Ubi4. As shown in Figure 5C-D, the deletion of *UBP3* robustly induced Ubi4 expression under both normal conditions and after 2-DG treatment. These results indicate that Ubi4 acts downstream of Ubp3, and that Ubp3 negatively regulates the level of Ubi4. Moreover, we found that Ubi4 protein expression was not affected by LCBs in the absence of Ubp3 (Figure 5E-F), suggesting an indispensable role for Ubp3 in the regulation of Ubi4 by LCBs. Finally, the deletion of *UBI4* in the *ubp3Δ* mutant resulted in a normal SG formation level compared to that in the WT (Figure 5G). Also, the Pab1 expression was not affected in the Ubp3 deletion strain (Figure S5C). Moreover, the changes in SG formation of *ubi4∆* and *ubp3∆* mutants are independent of PBs (Figure S5D-E). Last, we showed that the SG phenotype of *ubi4∆* and *ubp3∆* mutants is similar under arsenite (Figure S5F) as under 2-DG treatment, suggesting that the Ubi4 and Ubp3 may commonly regulate SG formation under stressful conditions in yeast cells.

Taken together, the above results suggest that, in the process of SG formation, LCBs function through suppressing Ubp3 expression to upregulate the level of Ubi4.

### Ubp3 and Ubi4 regulate Lsm7 foci formation

**Figure 6:**
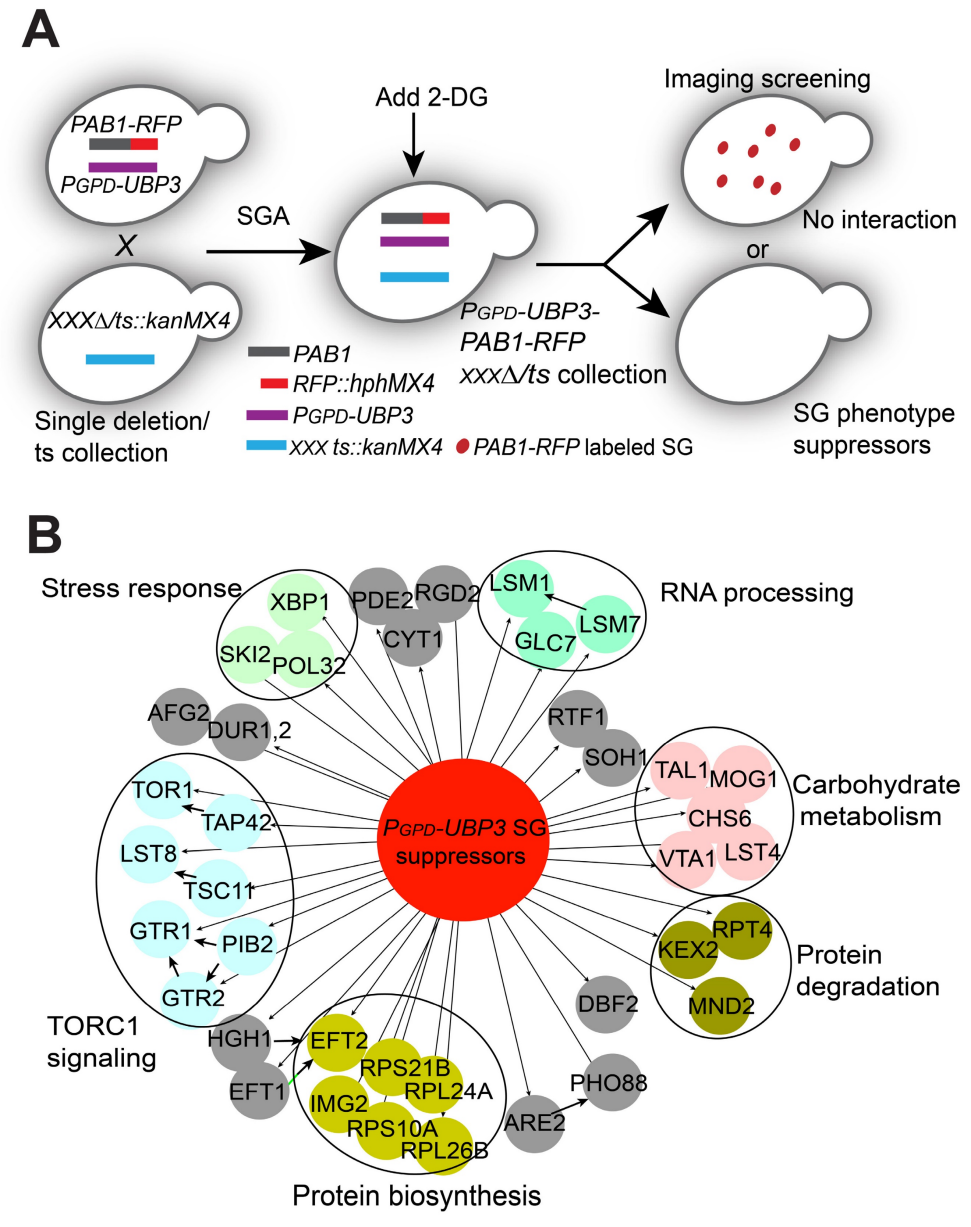
** A high-content screening-based study identified genes that suppress UBP3 overexpression-induced SG formation. A,** The workflow for screening *P_GPD_-UBP3-*mediated SG formation suppression. **B,** Network and functional analyses of the 45 *P_GPD_-UBP3* SG formation suppressors. Hits showing SG formation-suppressing effects (nodes linked with dark arrows) were grouped into modules based on their known physical interactions (bold dark arrows) and published cellular processing information. The functional groups are shown in different colored nodes. Dark circles indicate subunits or complexes within groups.

**Figure 7:**
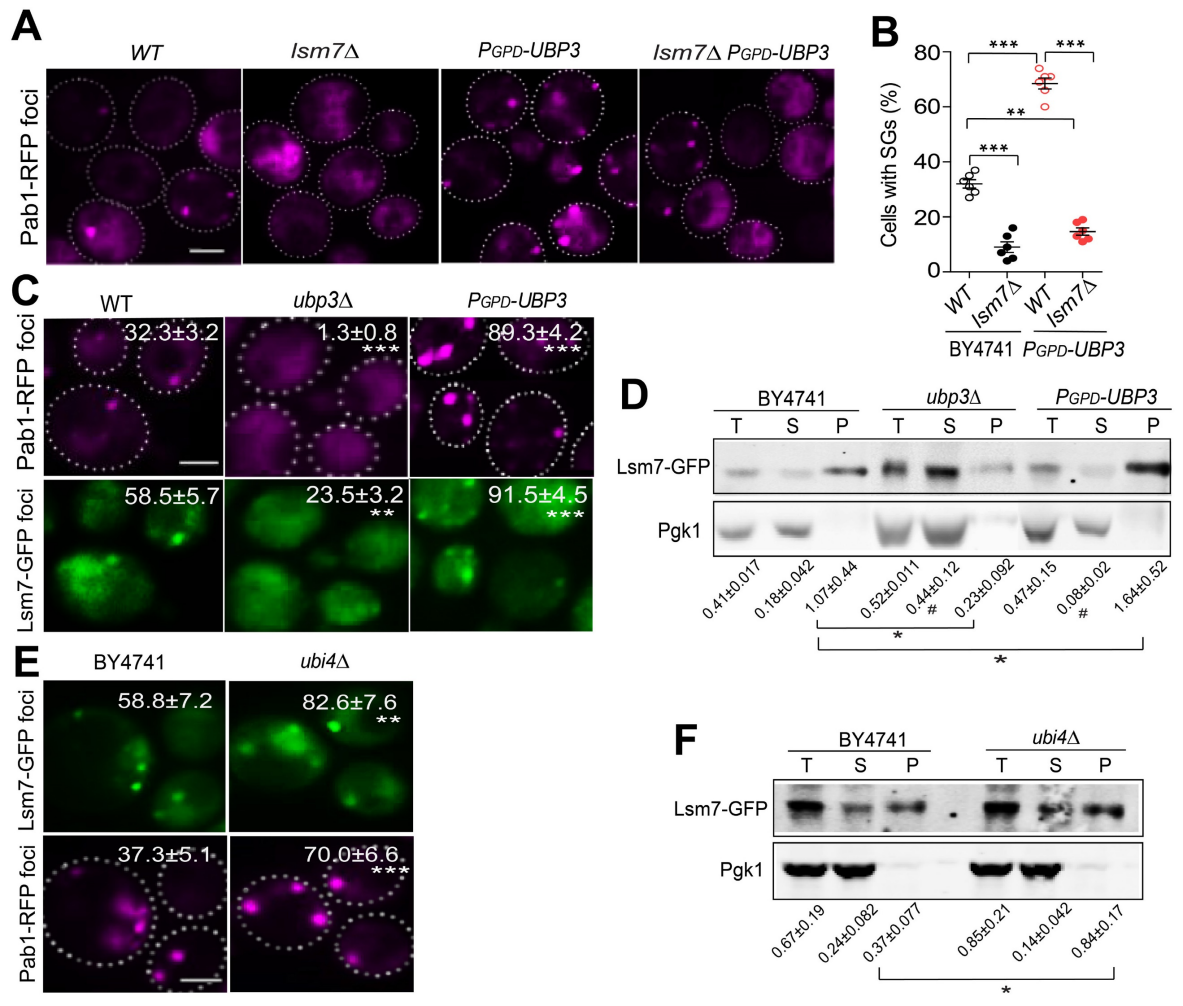
** Ubp3 and Ubi4 regulate Lsm7 phase separation. A-B,** Knockout of *LSM7* almost completely suppressed the increase in SG formation in the *P_GPD_-UBP3* strain. The bars in (**A**) show the means ± S.E.Ms. (n = 6). The circle represents the mean of individual clones. Six clones were examined for each strain, and >300 cells were analyzed for each clone. The scale bars indicate 2 µm, ** P < 0.01; *** P < 0.001 (one-way ANOVA followed by Tukey's test (**B**)). **C,** Lsm7-GFP aggregates were significantly decreased in the *ubp3∆* strain and increased in the *P_GPD_*-*UBP3* strain (upper panel, SGs; values represent the percentage of cells with SGs; lower panel, Lsm7 aggregates; values represent the percentage of cells with Lsm7 aggregates). **D,** Deletion of *UBP3* significantly reduced the insoluble form (P) and increased the soluble form (S) of the Lsm7 protein, while *UBP3* overproduction dramatically increased the insoluble form (P) and decreased the soluble form (S). The data are representative of three independent experiments. The values are presented as the means± SDs of arbitrary units (the intensities of the target bands were normalized to the Pgk1 levels in the corresponding fractions; for the pellet fractions, the values were normalized to the total Pgk1 level). * P < 0.05; (pellet fraction, one-way ANOVA followed by Dunnett's test). # P < 0.05 (soluble fractions, one-way ANOVA followed by Dunnett's test).** E,** Lsm7-GFP aggregates were significantly increased in the *ubi4∆* strain (upper panel, SGs; values represent the percentage of cells with SGs; lower panel, Lsm7 aggregates; values represent the percentage of cells with Lsm7 aggregates). (**C, E**) Four clones were tested for each strain, and 300 cells were analyzed for each clone. The results are presented as the means ± S.D.s. Scale bars indicate 2 µm. ** P < 0.01; *** P < 0.001 (one-way ANOVA followed by Dunnett's test).** F,** Deletion of *UBI4* increased the insoluble form (P) and decreased the soluble form (S). The data are representative of three independent experiments. The values are shown in **F.** * P < 0.05; (pellet fraction, one-way ANOVA followed by Dunnett's test).

Next, we aimed to explore the possible downstream targets/substrates of Ubi4 and/or Ubp3 in SG formation signaling. Since we found that cells with endogenous overexpression of *UBP3* had an increased SG formation phenotype (Figure S6), we decided to use the *UBP3* overexpressor to perform another imaging-based genome-wide screen to search for further downstream factors. We reasoned that the downstream targets of Ubi4 and Ubp3 should fulfill two criteria: 1) their null mutations should directly abolish/suppress the increase in SG formation induced by *UBP3* overproduction, and 2) they should belong to or be associated with critical SG structural components. Therefore, we performed a genome-wide phenotypic suppressor screen (Figure 6A) to identify the components that can suppress the increase in SG formation caused by Ubp3 overexpression (*P_GPD_-UBP3*). We isolated 45 suppressors that were enriched for processes related to (1) TOR complex 1 signaling, (2) RNA processing, (3) stress response, (4) protein degradation, and (5) carbohydrate metabolism (Figure 6B). The RNA processing group is of particular interest since one important protein, Lsm7, was recently reported to be a SG component and a key early phase separation factor that promotes the initiation of SGs under 2-DG treatment 70. A manual test confirmed the phenotype of the knockout of *LSM7* almost completely suppressing the increase in SG formation in the *UBP3* overexpression strains (Figure 7A-B). Therefore, we wanted to investigate whether and how Lsm7 was affected by Ubi4/Ubp3.

As reported, Lsm7 can undergo phase separation under 2-DG treatment, thereby promoting SG formation 70. We thus determined whether Lsm7 foci (phase-separated droplets) could be regulated by Ubp3 and Ubi4. We found that the number of Lsm7-GFP foci was significantly decreased in the *UBP3* deletion strain and increased in the *UBP3* overproducer strain (Figure 7C). These data suggest that Ubp3 may play a functional role in regulating Lsm7 phase separation. To test this, we first assessed the effect of Ubp3 on the solubility of Lsm7. We found that the deletion of *UBP3* significantly reduced the proportion of insoluble Lsm7 protein, while *UBP3* overproduction dramatically increased this proportion; and that the total level of Lsm7 was not altered (Figure 7D). Reciprocally, the deletion of Ubi4 enhanced the number of Lsm7 foci and the insoluble Lsm7 fraction (Figure 7E-F). Taken together, these results indicate that Ubp3 positively regulates the phase separation of Lsm7 by suppressing Ubi4, thereby promoting SG formation under 2-DG treatment.

### Sphingolipids metabolism pathway regulates SG formation in mammalian cells

**Figure 8:**
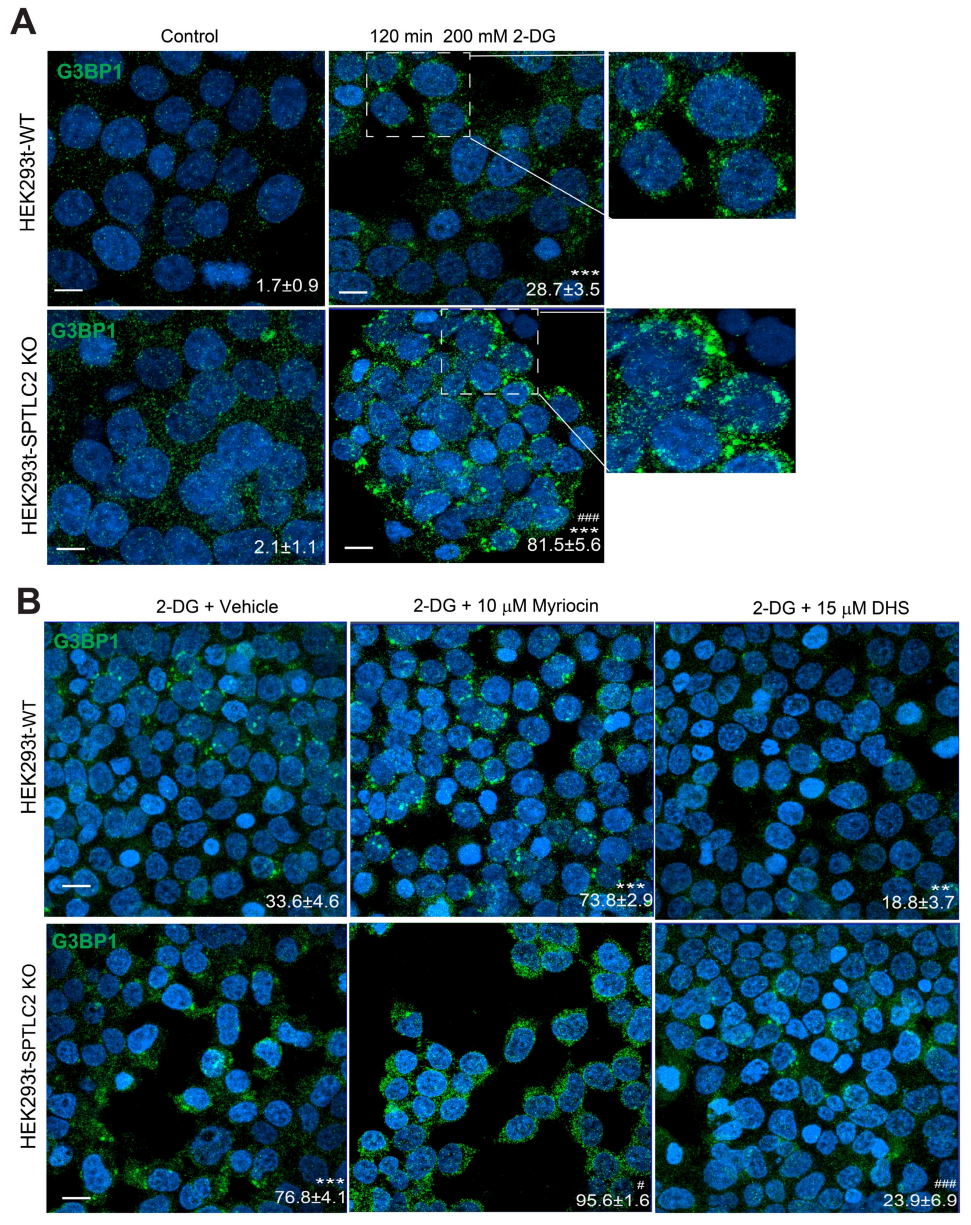
** Sphingolipids metabolism pathway regulates SG formation in mammalian cells. A,** SPTLC2 knockout HEK293t cells had increased SG formation (G3BP1 foci) under 2-DG treatment. Scale bars indicate 20 μm. Values represent the percentage of cells with SGs (G3BP1 foci), >300 cells were analyzed for each sample. Data were analyzed using the two-way ANOVA, *** P < 0.001 (2-DG vs Control); ### P < 0.001(SPTLC2 KO vs WT under 2-DG treatment). **B,** Myriocin induced, while DHS suppressed SG formation in the WT or SPTLC2 knockout HEK293t cells under 2-DG treatment. Scale bars indicate 20 μm. Values represent the percentage of cells with SGs (G3BP1 foci), >300 cells were analyzed for each sample. Data were analyzed using the two-way ANOVA, ** P < 0.01, *** P < 0.001 (vs WT treated with 2DG + Vehicle); # P < 0.05, ### P < 0.001 (vs SPTLC2 KO treated with 2-DG + Vehicle).

To further test whether our findings in yeast cells could be transferred to mammalian cells, we used wild-type and SPTLC2 (the human LCB2-like isoform) knockout HEK293t cells and G3BP1 as a marker for SGs. We found significantly increased SG formation under 2-DG treatment in wild-type cells, and SPTLC2 knockout enhanced this effect (Figure 8A). Furthermore, in both wild-type and SPTLC2 knockout HEK293t cells treated with 2-DG, myriocin significantly increased SG formation, whereas DHS suppressed SG formation (Figure 8B). These data demonstrated that the sphingolipid pathway regulates SGs formation in both yeast and mammalian cells.

**Figure 9:**
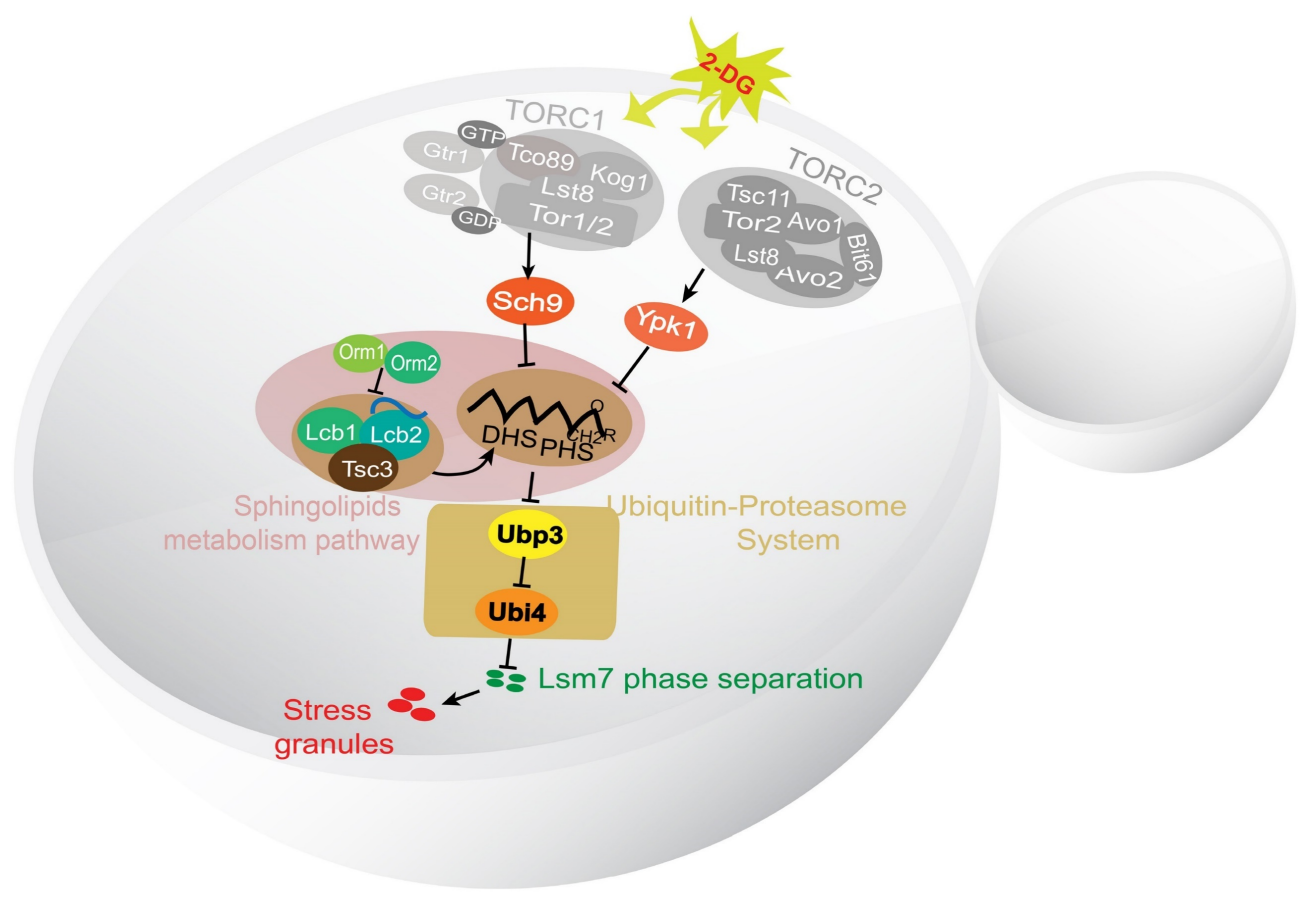
** A working model for the signaling pathway controlling the formation of SGs in yeast under 2-DG treatment.** A working model demonstrating the SG formation signaling pathway identified in this work. TORC1/2 was determined to be involved in an early step in this pathway. The signal was further transduced through its downstream effectors Sch9 and Ypk1 to influence the level of LCBs. Downstream of the LCBs, Ubi4 and Ubp3 together modulate Lsm7 phase separation and thereby regulate SG formation.

In summary, we have identified the major components of a potential signaling pathway controlling SG formation in yeast (Figure 9). Sch9 and Ypk1 were found to be involved in this pathway downstream of TORC1/2. The stress signal was transduced through Sch9 and Ypk1 to result in a decreased level of LCBs in the cell. The decreased LCBs caused an increase in the level of Ubp3, and the higher Ubp3 expression further represses the expression of Ubi4, the lowered Ubi4 level stimulates the phase separation of Lsm7, which in turn seeds the formation of SGs.

## Discussion

In this study, we have identified the major components of a signaling pathway controlling the formation of SGs in yeast under 2-DG treatment (Figure 9). We show that the TORC1/2-Sch9/Ypk1 signaling cascade regulates SG formation mainly through modulating the major long-chain base sphingolipids (LCBs) in the cell. Further we demonstrate that the ubiquitin-proteasome system components Ubi4 and Ubp3 initiate the phase separation of Lsm7, which later triggers SG formation.

Sphingolipids have been linked to stress responses because they mediate transcriptional reprogramming, translation of heat-shock proteins, and protein quality control 36, 63. Our observation of the suppression of SG formation by LCBs could explain the proapoptotic properties of these factors since SG formation prevents cell apoptosis not only by inhibiting translation initiation but also by sequestering certain apoptotic factors 71. Therefore, the inhibition of SG formation may release apoptotic regulatory factors that trigger apoptosis or result in the inability to restrict the accumulation of ROS, which is a key inducer of apoptosis 43. These reports, together with our results, suggest that sphingolipids play a wide role in stress responses and cell fate by modulating SG formation.

Data from this work suggest that the LCB sphingolipids may serve as messengers to regulate Ubi4 expression via Ubp3 under stress. These results may help us understand the mechanism by which the depletion of ubiquitin results in neuronal cell death and further neurodegenerative diseases. The ubiquitin pathway is highly active in synapses 72, and neurons are susceptible to ubiquitin depletion under stress 73, 74. Furthermore, the depletion of ubiquitin may lead to persistent SG formation under stress, which can sequester some functional proteins or impair ribostasis and cause other pathological changes in neurons 23, 75. Additionally, a recent study has shown that the number of ubiquitin units encoded by *UBI4* influences proteostasis and stress survival and that the optimal *UBI4* repeats vary under different stress conditions 76, suggesting an important role for ubiquitin during eukaryotic stress adaptation. However, whether SG formation is involved in this evolutionary alternative has yet to be determined.

SGs colocalize with free ubiquitin but not polyubiquitinated proteins 38, which may suggest a potential role of free ubiquitin in the regulation of SG assembly. Moreover, SGs are formed through LLPS of certain proteins and RNA, where ubiquitin and polyubiquitin have been shown to eliminate the phase separation behavior of SG components 39. This may further suggest that free ubiquitin could affect SG formation by influencing the LLPS of SG constituents. Accordingly, a recent study reported that Lsm7, a component of SGs, undergoes phase separation upon 2-DG treatment, which subsequently induces SG formation 70. Our observations further provide evidence that Ubp3 positively regulates the phase separation of Lsm7 via the suppression of Ubi4 expression, thereby promoting SG formation under 2-DG treatment. Nevertheless, the existence and possible functions of other components that participate in this interaction between Ubp3, Ubi4, and Lsm7 need to be elucidated.

Here, we report key components in a possible pathway that controls SG formation under 2-DG- and arsentie-induced stress conditions. This pathway might represent a mechanism for SG formation under energy/nutrient depletion and oxidative stress conditions, where much of the microbial biomass in the world is believed to exist 77. Furthermore, we have shown that the pathway regulating SG formation is also conserved in mammalian cells. However, the functions of these key SG regulators need to be further investigated in mammalian model systems. Such studies will improve our understanding of the mechanisms underlying SG-induced drug resistance and the relationship between SGs and age-related diseases such as cancer and neurodegeneration.

## Methods

### Cell culture

All the strains used in this study were from the BY4741/4742 or SGA (S288C) background (the strains are listed in Table S2) and were grown at 30 °C or at the indicated temperatures. Yeast rich medium (YP) containing 1% Bacto yeast extract and 2% Bacto peptone was supplemented with 2% glucose (YPD). Yeast minimal medium (YNBD) contained 0.67% Difco yeast nitrogen base without amino acids and 2% glucose. Supplements essential for auxotrophic strains were added to 20 mg/L for bases and amino acids (complete) except for leucine (SC-Leu), histidine (SC-His) or uracil (SC-Ura). In this study, the *his3∆* strain was used as the wild-type control strain for the generation of mutants via the SGA approach.

### Yeast strain construction

Single, double, or triple null mutants were constructed by PCR amplification and insertion with selective markers, including *LEU2*, *natMX4*, and *kanMX4* (primers are listed in Table S3). The strains used for protein expression and localization analyses were picked directly from the yeast GFP collection or constructed by using standard PCR to integrate an *Aequorea victoria* GFP (S65T) tag into the yeast chromosome (C-terminus of the ORF) through homologous recombination, after which the constructs were expressed using endogenous promoters 78. The *UBP3* overexpression strain PGPD-UBP3 was constructed by amplifying the natNT2-containing cassette from the pYM-N15 plasmid using specific primers (Table S3) and then inserting it into the upstream promoter region of *UBP3* by homologous recombination.

### Screening for SG formation in temperature-sensitive (ts) alleles

To efficiently incorporate the SG marker (Pab1-RFP) into the yeast ts collection (TS-V5), the yeast synthetic genetic array (SGA) methodology was applied to combine the Pab1-RFP marker into a single haploid cell through standard mating and meiotic recombination via a robotic SGA procedure using the Singer RoToR HDA system (Singer Instruments) 46, 48. The screening and manual confirmation were performed as described previously, with some modifications 47. Briefly, the ts mutants were precultured at 22 °C for 2-3 days, diluted to an OD_600_ of 0.2 in SD media and grown with shaking at 30 °C until an OD_600_ of 0.6 was reached. The cells were then treated with 400 mM 2-DG for 2 h, fixed and washed. Images were acquired in an automated cellular imaging and analysis system (ImageXpress MICRO (MDC)). The SG formation in the mutants was quantified by MetaXpress (version 3.1) software by using the MetaXpress subprogram. For manual quantification, the cells were precultured at 22 °C for 2 days, diluted and grown at 30 °C to an OD_600_ of 0.5 before the application of 2-DG, formaldehyde fixed and washed as described above. The SG phenotype was microscopically studied (Zeiss AxioObserver. Z1, Germany) and quantified (the ratio of cells with Pab1-RFP foci) using ImageJ software with the cell-counter plug-in.

### Genome-wide high-content screening for *P_GPD_-UBP3 suppressors*

To construct the null/ts allele collection with *UBP3* overexpression, *P_GPD_-UBP3* and Pab1-RFP were both introduced into the yeast query strain Y7039 (*MATα*,* can1∆::STE2pr-Sp_Ura3 lyp1∆*,* his3∆*,* leu2∆*,* ura3∆*,* met15∆*). The collection was then constructed using the SGA method described above. The SG formation in this collection was then screened and manually confirmed as described above with some modifications (precultured in SGA final media [SD-Leu/Arg/Lys + S-AEC, Canavanine, and Hygromycin B] at 30 °C for 2 days).

### Functional enrichment and interaction network analysis

The functional enrichment of the confirmed hits from the SG formation screen (ts alleles library) was tested using the BiNGO plug-in for Cytoscape (Cytoscape v3.2.1) 49. The Gene Ontology biological process term with the highest enrichment in a particular cluster was used to label the cluster on the network. The top hits were also analyzed by using the Gene Ontology Term Finder 50 for enrichment of Gene Ontology (GO) biological processes, molecular functions, and cellular component categories by comparison with a background set of genes (the top 60% of the ts alleles [TS-V5] that show an effect on growth rate at 30 °C; listed in Li *et al.* 46). A cutoff of P < 0.05 was used. For the screens for SG components, the interaction network diagram of the hits was extracted from the interaction analysis using Osprey 1.2.0, and the physical interactions among confirmed hits were added according to the BioGRID interaction database.

For the screens for SG components and for* P_GPD_-UBP3* SG suppressors, the interaction network diagram of the hits was constructed via interaction analysis using Osprey 1.2.0, and the physical interactions among the confirmed hits were added to the BioGRID interaction database. The top hits from the *P_GPD_-UBP3* SG suppressor screen were also analyzed using the Gene Ontology Term Finder for enrichment of Gene Ontology (GO) biological processes by comparison with a background set list representing TS-V5 plus SGA-V2 and an array of slow-growing mutants. A cutoff of P < 0.05 was used.

### SG induction with 2-DG

For the two screens described above, cells were grown in 96-well plates with a starting OD_600_ of 0.2. When the OD_600_ reached 0.6, the cells were treated with 2-DG for 2 h at a final concentration of 400 mM to induce SG formation.

### Treatment with myriocin, DL-dihydrosphingosine and phytosphingosine hydrochloride

To study the SG phenotype resulting from SPT complex inhibition, cells were grown with a starting OD_600_ of 0.1 in the presence of a vehicle (methanol) or 600 ng/mL myriocin (Sigma, M1177). The cells were treated with 400 mM 2-DG for 2 h until the OD_600_ reached 0.5 (5 h were needed with methanol alone but 9 h were needed with myriocin), after which the cells were fixed with 3.7% formaldehyde and washed twice with PBS. To study the sphingolipid synthesis pathway, DL-dihydrosphingosine (DHS; Sigma, D6783) and phytosphingosine hydrochloride (PHS; Sigma, P2795) were used to treat cells as described above for myriocin treatment.

### Fluorescence microscopy

The cells were grown to an OD_600_ of 0.5 with or without treatment as described above. The cells were then fixed with 3.7% formaldehyde for 30 min and washed twice with PBS. A Zeiss Axiovert 200 M fluorescence microscope was used to obtain images using the GFP and RFP channels.

### Treatment with arsenite

The cells were cultured to an OD_600_ of 0.5. Sodium arsenite (NaAsO2; Sigma-Aldrich, St. Louise, MO, USA) was added to cell cultures at the concentration of 2 mM for 3 h. The cells were then fixed with 3.7% formaldehyde for 30 min and washed twice with PBS. A Zeiss Axiovert 200 M fluorescence microscope was used to obtain images using the RFP channel.

### Time-lapse imaging assay

The cells were incubated to an OD_600_ of 0.5. The observation was started right after when the cells were treated with 400 mM 2-DG and transferred to 0.25 mg/mL concanavalin A coated 96-well plate. Images were acquired in an automated cellular imaging and analysis system (ImageXpress MICRO (MDC)) using the RFP channel with an interval of 3 min for 6 h. The SGs fusion time was studied by ImageJ software. 100 SGs fusion cases were measured in each strain. The fusion time was calculated as the duration between the two SGs observed until they fuse into one stable SG.

### Lipidomics analysis

Indicated yeast cells were cultured in SD medium to log phase, and then treated with or without 400 mM 2-DG for another 2 h. About 7 OD cells were collected to 1.5 mL centrifuge tube and were immediately snap-frozen in liquid nitrogen and transported on dry ice for lipidomic analysis.

The yeast lipidomics analysis was conducted at LipidALL Technologies as described previously^79^. Briefly, lipid extraction was performed by homogenizing yeast cells in 750 µL of chloroform:methanol 1:2 (v/v) containing 10% deionized water with glass beads on an automated bead shaker. The homogenate was then incubated at 4 °C for 1 h, followed by the addition of 350 µL deionized water and 250 µL chloroform. The lower organic phase containing lipids was extracted by centrifugation and was further dried in the SpeedVac under OH mode. Samples were stored at -80 °C until further analysis.

Lipidomics methodology was reported according to standard guidelines. Polar lipids PC, LPC, Sph, and PhytoCer were analyzed in the ESI positive ion mode on Agilent 1260 HPLC coupled to Sciex 5500 QTRAP, with source parameters CUR 10, TEM 400 °C, GS1 30, GS2 30. Spiked internal standards, including d9-PC32:0 (16:0/16:0), d7-PE33:1 (15:0/18:1), d31-PS, d7-PG33:1 (15:0/18:1), d7-PI33:1 (15:0/18:1), d7PA33:1 (15:0/18:1), Cer (d18:1-d7/15:0), d7-LPC18:1, d7-LPE18:1, C17:0-LPA, C17:1-LPI, C17:1-LPS, d17:1-Sph (Avanti Polar Lipids. Inc.) were used to quantify individual lipid species.

### Western blotting

The total yeast protein extraction method 80 was used with modifications. Approximately 1.0 unit OD_600_ of yeast cells was harvested and incubated in 1 mL of 0.2 M NaOH for 20 min on ice. The mixture was subsequently resuspended in 50 µL of HU sample buffer (8 M urea, 0.2 M Tris-HCl (pH 6.8), 1 mM EDTA, 5% SDS, 1% β-mercaptoethanol, and 0.0025% bromophenol blue) and heated for 10 min at 70 °C. All samples were electrophoresed on 10% Tris-HCl/SDS-polyacrylamide gels (Bio-Rad) and transferred onto polyvinylidene difluoride membranes (Merck Millipore). The membranes were then incubated with primary antibodies (mouse anti-Pgk1 (Invitrogen, #459250), rabbit anti-GFP (Abcam, ab6556), or anti-RFP (Abcam, ab62341)) followed by secondary antibodies (goat anti-mouse IRDye^®^ 680RD (LICOR, IR Dye 926-68180) or goat anti-rabbit IRDye^®^ 800CW (LICOR, IR Dye 926-32210)). Relative protein expression was analyzed by an Odyssey^®^ imaging system (LICOR) and was normalized to that of Pgk1.

### Protein aggregation assay

Soluble and insoluble (aggregate) proteins were extracted as described previously 81. Yeast cells were grown to an OD_600_ of 0.6-0.8 and harvested by centrifugation. Approximately 10 units of OD_600_ cells were resuspended in 300 μL of 50 mM Tris-HCl (pH 8.5)/500 mM NaCl/1 mM phenylmethylsulfonyl fluoride (PMSF)/EDTA-free protease inhibitors (Roche). The cells were pulverized with a vortex mixer and glass beads (diameter 0.5 mm; Biospec) and were precleared by centrifugation at 3,000 × g for 5 min. A 200-μL volume of the supernatant was taken, and 90 μL was stored as total protein. The remaining 110 μL, which was used for fractionation of soluble and insoluble proteins, was centrifuged at 16,000 × g for 20 min. The resulting supernatant contained the soluble fraction. The pellet, which contained the insoluble fraction, was washed in 110 μL of 50 mM Tris-HCl (pH 8.5)/150 mM NaCl/1 mM PMSF/EDTA-free protease inhibitors (Roche), centrifuged at 16,000 × g for 20 min, and resuspended in 110 μL of 8 M urea/2% SDS/50 mM Tris (pH 8.5)/150 mM NaCl/1 mM PMSF/2 mM dithiothreitol/EDTA-free protease inhibitors (Roche). The same volumes of soluble and insoluble fractions were used for quantitative western blotting. To calculate the soluble or insoluble fraction of a protein, the signal of the supernatant or pellet was divided by the total signal of the supernatant and pellet of the same sample.

### SG formation examination in mammalian cells

The SPTLC2 knockout cell line (HEK293t) was constructed as described previously 36. Cells were cultured in Dulbecco's modified Eagle's medium (Gibco, c11995500bt) supplemented with 10% fetal bovine serum (FBS, ExcellBio) and 1% penicillin-streptomycin (HycloneTM, SV30010) in a humidified incubator containing 5% CO_2_ at 37 °C.

For SG formation examination studies, cells were seeded onto confocal imaging plates (Nest, 801002) and treated with 10 μM myriocin or 15 μM DHS when the cells had reached 90% confluency. 200 mM 2-DG was added to induce SG formation (2 h). Cells were fixed with 4% paraformaldehyde for 15 min and rinsed in PBS for further use.

For immunofluorescent studies, fixed cells were permeabilized with 0.5% Triton X-100 in PBS for 5 min, and blocked with 10% donkey serum for 1 hour. Samples were further incubated with G3BP1 (Proteintech 13057-2-AP) in blocking buffer at 4 °C overnight. Samples were washed three times with PBS and incubated with goat anti rabbit Alexa Fluor 488 (Thermo Fisher Scientific) for 2 h at room temperature, followed by DAPI (Thermo Fisher Scientific P36931) staining for 15 min. Images were captured and visualized using a Leica DMI4000 fluorescence microscope.

### Statistical analysis

No statistical methods were used to predetermine the sample size. Appropriate statistical analyses were performed depending on the comparisons made in the text and figure legends. One-way or two-way ANOVA following Dunnett's test, Tukey's test, or two-tailed unpaired Student's t test was performed using Prism version 8 (GraphPad, Inc.). P values are designated *P < 0.05, **P < 0.01, and ***P < 0.001. All the graphs show the mean and error bars, which represent the standard error of the deviation (S.D.) or the standard error of the mean (S.E.M.).

## Supplementary Material

Supplementary figures.

Supplementary table.
