# Supplementary material for: Stress granule formation is regulated by signaling machinery involving Sch9/Ypk1, sphingolipids, and Ubi4
Source: Theranostics. 2025 Jan 6;15(5):1987–2005. doi: 10.7150/thno.98199 (PMC11780528; doi:10.7150/thno.98199)
Supplement: Supplementary file 1 — Supplementary figures. [file thnov15p1987s1.pdf]

Supplementary figures

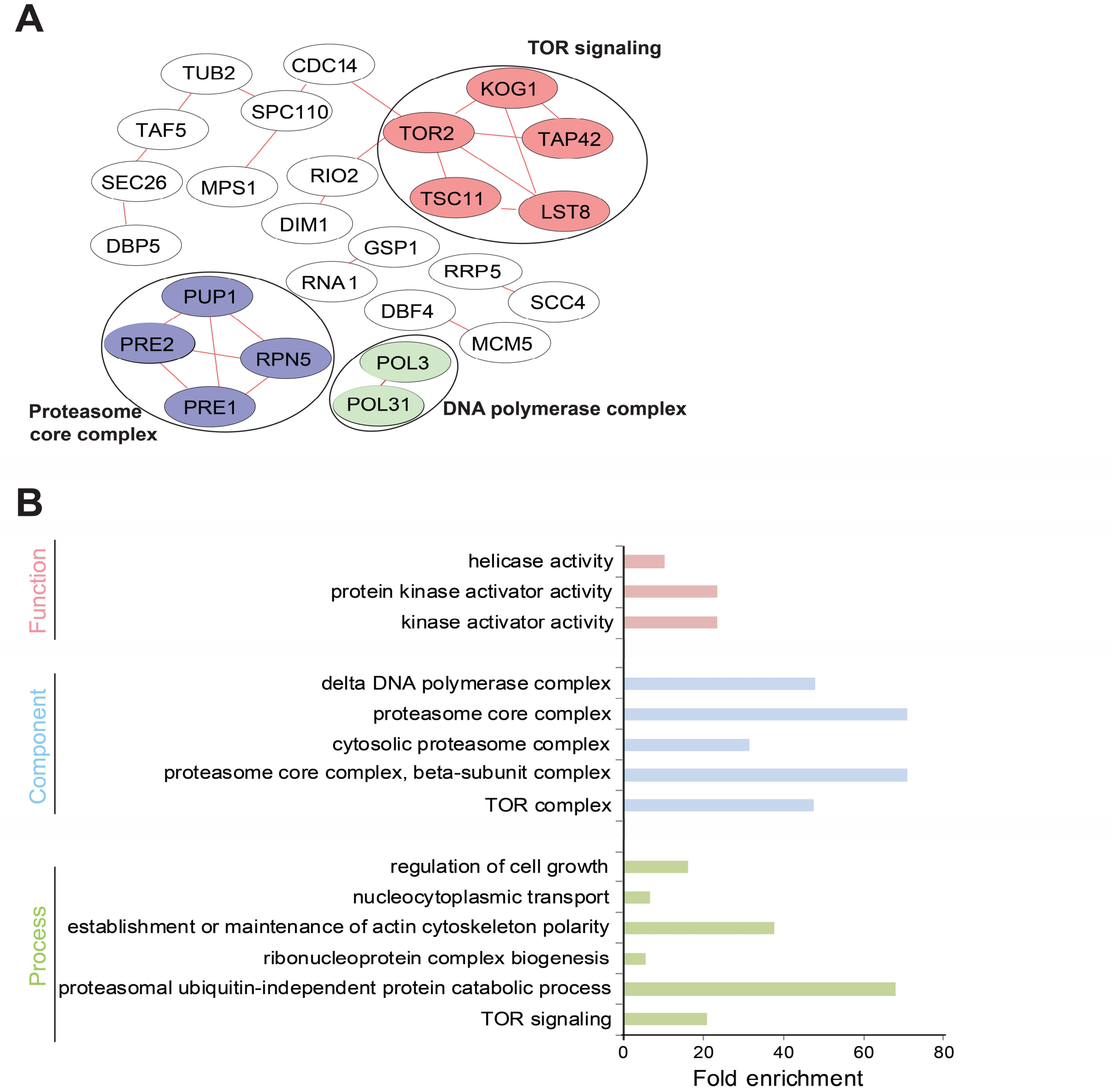

Figure S1. Functional enrichment analyses for the ts alleles associated with decreased SG formation

A, Functional enrichment analysis of the ts alleles associated with decreased SG formation. Mutants with confirmed SG phenotypes were analyzed for enrichment of the GO biological process, function and component categories. Enriched groups were scored by comparison to the essential gene temperature-sensitive allele collection (top 60% of the ts alleles that showed an effect on growth rate at 30 °C) using a cutoff of P < 0.05. **B,** Functional enrichment analysis of the ts alleles associated with decreased SG formation was performed as described in (**A**).

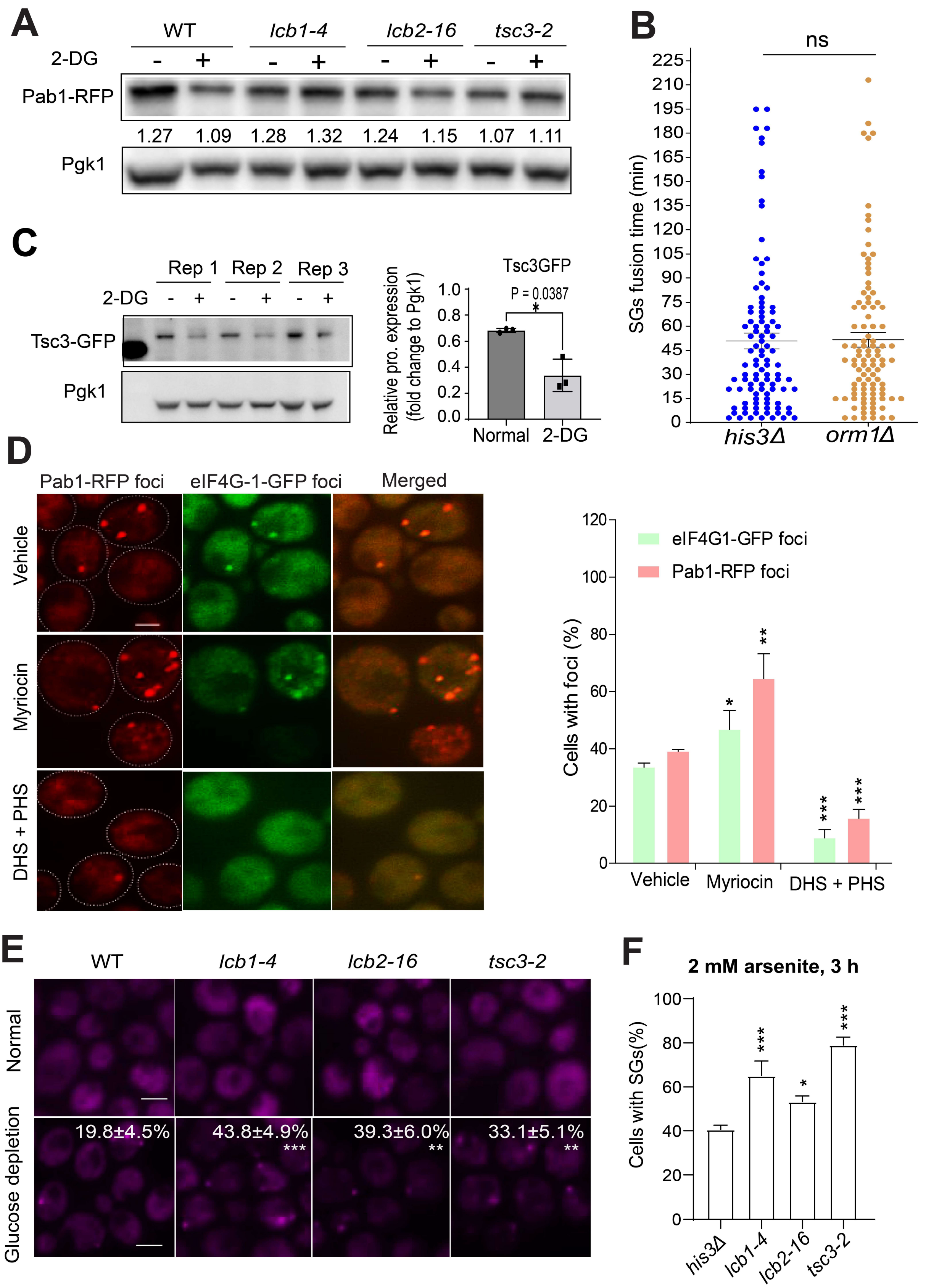

Figure S2. The SPT ts alleles had similar SG phenotype under glucose depletion and arsenite treatment, the fusion time of SGs was not changed in the Orm1 null mutant, the SPT component

tsc3 expression decreased under 2-DG, and the comparison between Pab1-RFP foci formation and eIF4G1 foci formation

A, Pab1-RFP protein expression levels in the wild type (WT) and in the SPT ts mutants under normal conditions or 2-DG treatment. The data are representative of three independent experiments. The values are presented as the means of arbitrary units (the intensity of the target bands was normalized to the Pgk1 level) for each clone. **B,** Time-lapse imaging assay was performed to determine the fusion time of SGs in WT or Orm1 null mutant under 2-DG treatment. 100 cases were measured in each strain. The time was calculated as the duration between the two SGs observed until they fuse into one stable SG. Ns, non-significant (Student's t-test). **C,** Tsc3-GFP protein expression levels in the wild type (WT) strain under normal conditions or 2-DG treatment. The values are presented as the means of arbitrary units (the intensity of the target bands was normalized to the Pgk1 level) for each clone (Student's t-test). **D,** Pab1-RFP foci formation and eIF4G1 foci formation treated with myriocin or DHS + PHS were determined and compared. The values represent the percentage of cells with foci. Three clones were examined for each strain. The data are presented as the means ± S.D. Scale bars indicate 2 μm. * $P < 0.05$, *** $P < 0.001$ (One-way ANOVA followed by Dunnett's test, treatments vs. Vehicle). **E-F,** Glucose depletion or arsenite treatment induced SG formation in the SPT ts alleles. The values or bars represent the percentage of cells with SGs and are shown as the means ± S.D. Three clones were examined for each strain, and >300 cells were analyzed for each colony. The scale bar indicates 2 μm, * $P < 0.05$, ** $P < 0.01$, *** $P < 0.001$ (One-way ANOVA followed by Dunnett's test).

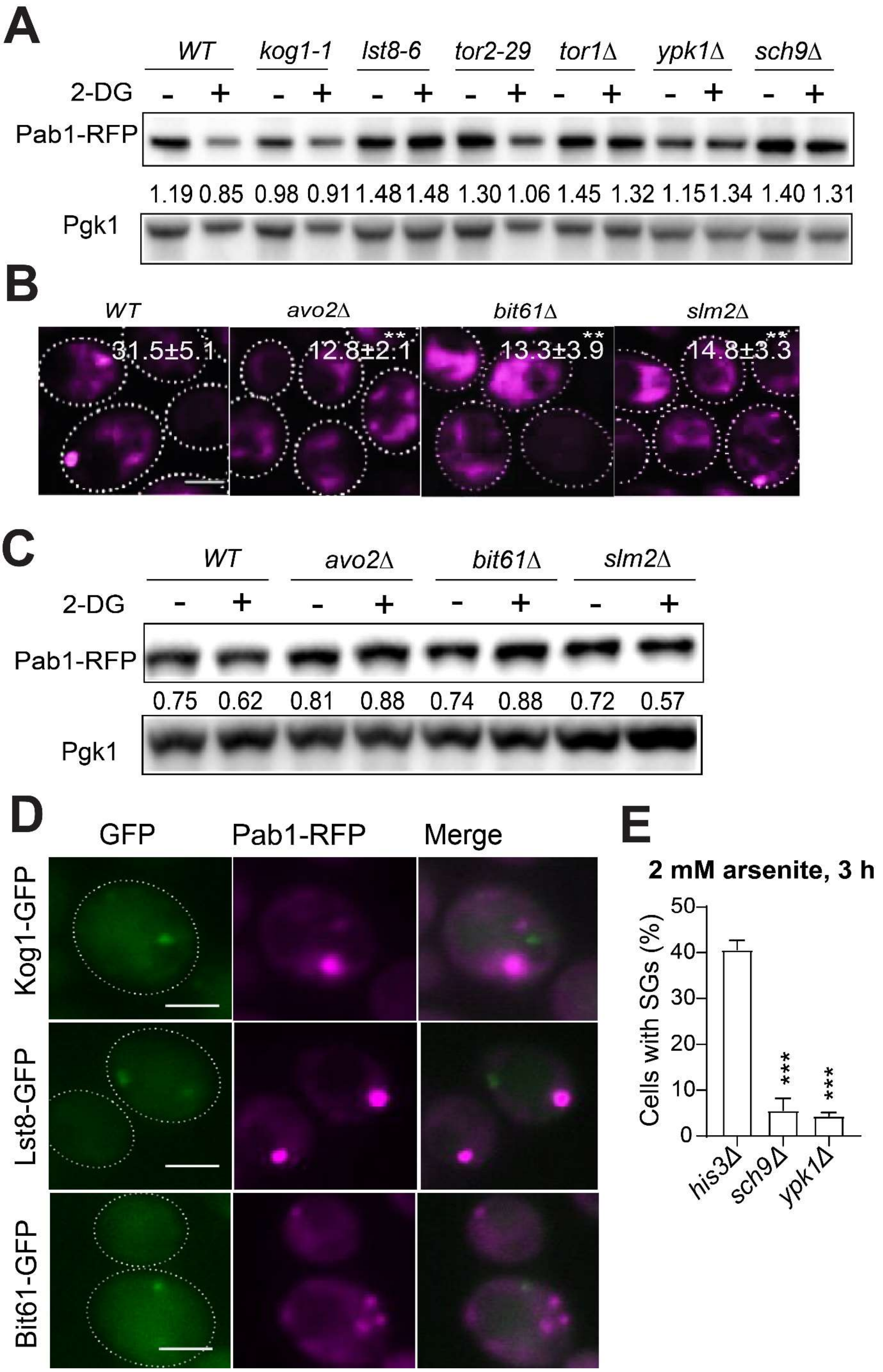

Figure S3. Mutants of the core components of TORC1/2 did not alter the Pab1-RFP expression level, no colocalization was observed between Pab1-RFP and these components (Kog1, Lst8 and Bit61), and the SPT ts alleles had similar SG phenotype under arsenite treatment

A, Pab1-RFP protein expression levels in the wild type (WT) and in the ts mutants or null mutants of the TORC1/2 core components under normal conditions or 2-DG treatment. The data are representative of three independent experiments. The values are presented as the means of arbitrary units (the intensity of the target bands was normalized to the Pgk1 level) for each clone. **B,** Deletion of other components of TORC2 (*AVO2*, *BIT61*, and *SLM2*) decreased SG formation. The values represent the percentage of cells with SGs and are shown as the means ± S.D.s. Four clones were examined for each strain, and >300 cells were analyzed for each colony. The scale bar indicates 2 μm, ** $P < 0.01$ (unpaired two-tailed Student's t test). **C,** Same as in (**A**), Pab1-RFP protein expression in the wild-type (WT) and null mutant TORC2 (*AVO2*, *BIT61*, and *SLM2*) strains under normal conditions or 2-DG treatment. **D,** The Kog1-GFP (upper), Lst8-GFP (middle), and Bit61-GFP (bottom) signals did not colocalize with SGs. The scale bars indicate 2 μm. The data are representative of two independent experiments. **E,** Arsenite treatment decreased SG formation in the Sch9 and Ypk1 null mutants. The bars represent the percentage of cells with SGs and are shown as the means ± S.D. Three clones were examined for each strain, and >300 cells were analyzed for each colony. *** $P < 0.001$ (One-way ANOVA followed by Dunnett's test).

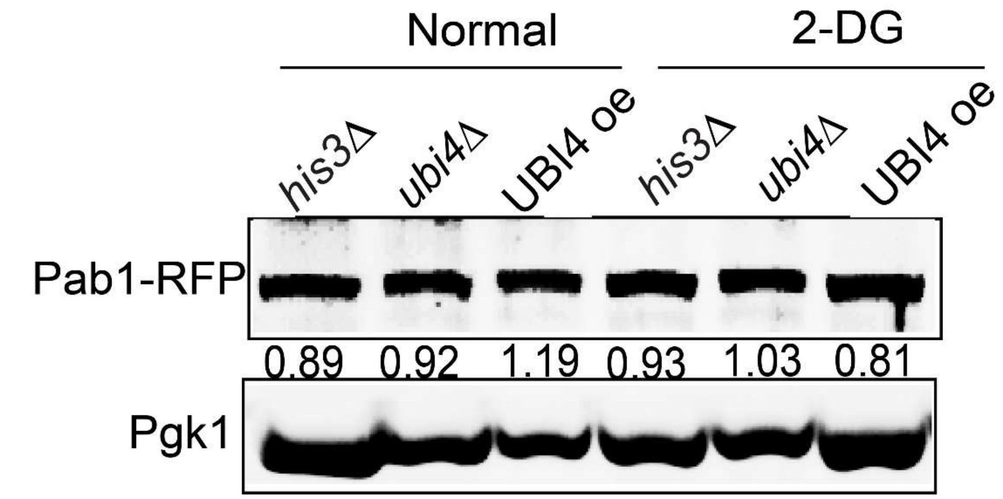

Figure S4. Pab1 expression was not changed in the Ubi4 deletion or overexpression strains

Pab1-RFP protein expression levels in the wild type (WT), the *ubi4*Δ mutant and the UBI4 overexpressing strain were determined as described in Figure S3A.

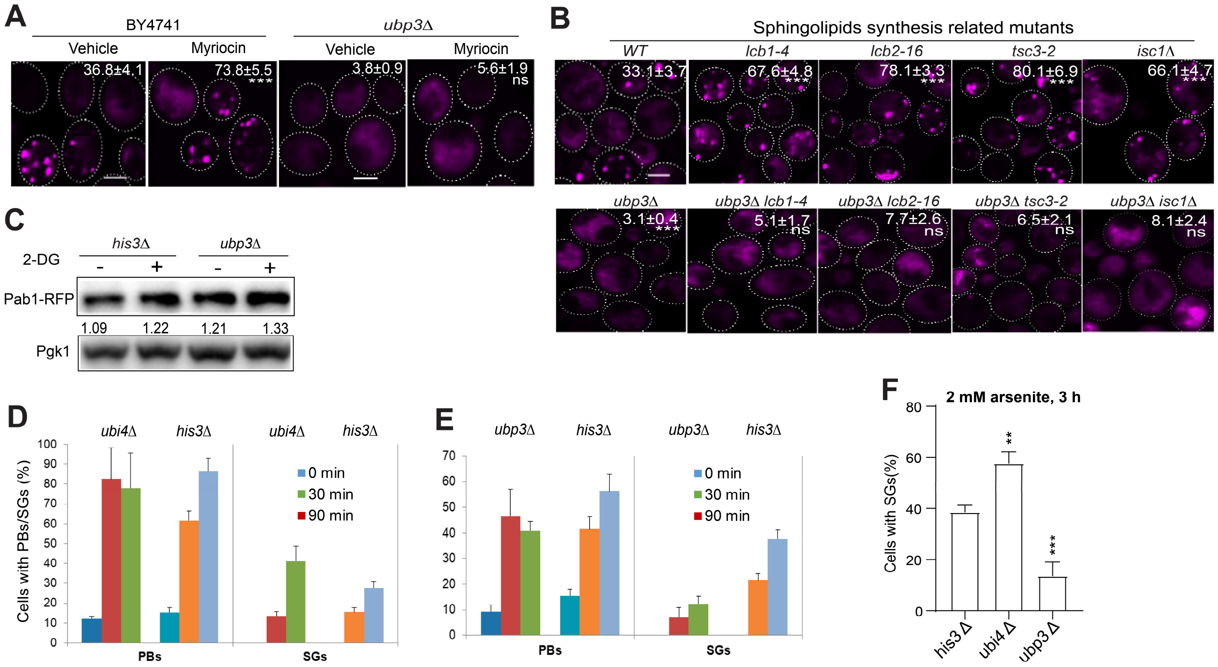

Figure S5. Ubp3 works downstream of LCBs and the changes in SG formation of *ubi4*Δ and *ubp3*Δ mutants are independent of PBs

A, Myriocin cannot induce SG formation in the absence of *UBP3*. The values represent the percentage of cells with SGs. **B,** Deletion of *UBP3* blocks the SG phenotypes of the *lcb1-4*, *lcb2-16*, *tsc3-2*, and *isc1*Δ mutants. The values represent the percentage of cells with SGs. ns indicates no significance (two-way ANOVA followed by Dunnett's test, double mutants vs. *ubp3*Δ); *** P < 0.001 (two-way ANOVA followed by Dunnett's test, double mutants vs. WT). (**A-B**) Four clones were examined for each strain. The data are presented as the means ± S.D. Scale bars indicate 2 μm. **C,** Pab1-RFP protein expression levels in the wild type (WT) and the *ubp3*Δ mutant were determined as described in Figure S3A. **D-E,** Influence of *UBI4* or *UBP3* deletion on PBs formation in the WT (*his3*Δ Pab1-RFP DCP2-GFP), the *ubi4*Δ (*ubi4*Δ Pab1-RFP DCP2-GFP) or the *ubp3*Δ (*ubp3*Δ Pab1-RFP DCP2-GFP) strains. Representative data from three independent experiments are shown as the mean ± S.D. **F,** Arsenite treatment induced SG formation in the Ubi4 and decreased SG formation in the Ubp3 null mutants. The bars represent the percentage of cells with SGs and are shown as the means ± S.D. Three clones were examined for each strain, and >300 cells were analyzed for each colony. ** P < 0.01, *** P < 0.001 (One-way ANOVA followed by Dunnett's test).

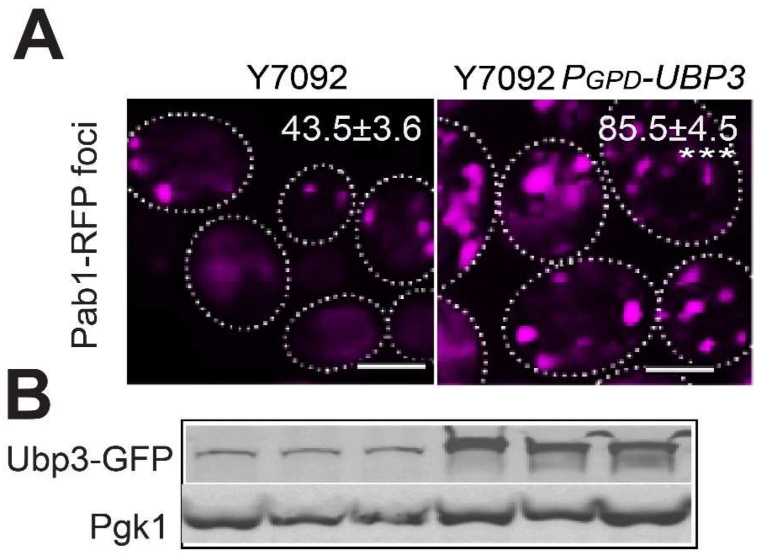

Figure S6. SG phenotype of the query strain Y7092 overexpressing Ubp3

A, The SG formation rate was significantly increased in the P_{GPD}-*UBP3* strain. The values represent the percentage of cells with SGs. **B,** Western blotting to confirm the overexpression of Ubp3 by using the GPD promoter in the query strain Y7092. Three clones were examined for each strain. The data are presented as the means ± S.D.s. Scale bars indicate 2 μm.
